# Identification of LINE retrotransposons and long non-coding RNAs expressed in the octopus brain

**DOI:** 10.1186/s12915-022-01303-5

**Published:** 2022-05-18

**Authors:** Giuseppe Petrosino, Giovanna Ponte, Massimiliano Volpe, Ilaria Zarrella, Federico Ansaloni, Concetta Langella, Giulia Di Cristina, Sara Finaurini, Monia T. Russo, Swaraj Basu, Francesco Musacchia, Filomena Ristoratore, Dinko Pavlinic, Vladimir Benes, Maria I. Ferrante, Caroline Albertin, Oleg Simakov, Stefano Gustincich, Graziano Fiorito, Remo Sanges

**Affiliations:** 1 Department of Biology and Evolution of Marine Organisms, Stazione Zoologica Anton Dohrn, Villa Comunale, SZN, 80121 Naples, Italy; 2 Institute of Molecular Biology (IMB), Mainz, Germany; 3 Central RNA Laboratory, Istituto Italiano di Tecnologia (IIT), Via Enrico Melen 83, 16152 Genova, Italy; 4 Department of Biomedical and Clinical Sciences, Linköping University, Linköping, Sweden; 5 Institute of Zoology, University of Cologne, Cologne, Germany; 6 Neurobiology Sector, Scuola Internazionale Superiore di Studi Avanzati (SISSA), Via Bonomea 265, 34136 Trieste, Italy; 7 Department of Integrative Marine Ecology, Stazione Zoologica Anton Dohrn, Villa Comunale, SZN, 80121 Naples, Italy; 8 Strand Life Sciences, Bengaluru, India; 9 Scientific Core Facilities & Technologies, GeneCore, European Molecular Biology Laboratory (EMBL), Meyerhofstrasse 1, 69117 Heidelberg, Germany; 10 Institute of Molecular and Clinical Ophthalmology, Basel, Switzerland; 11 Marine Biological Laboratory (MBL), Woods Hole, MA USA; 12 Okinawa Institute of Science and Technology Graduate University, Onna, Okinawa, 9040495 Japan; 13 Department of Molecular Evolution and Development, Wien University, Althanstraße 14 (UZA I), 1090 Wien, Austria

**Keywords:** Mollusks, Nervous system, Transcriptome, Transposable elements

## Abstract

**Background:**

Transposable elements (TEs) widely contribute to the evolution of genomes allowing genomic innovations, generating germinal and somatic heterogeneity, and giving birth to long non-coding RNAs (lncRNAs). These features have been associated to the evolution, functioning, and complexity of the nervous system at such a level that somatic retrotransposition of long interspersed element (LINE) L1 has been proposed to be associated to human cognition. Among invertebrates, octopuses are fascinating animals whose nervous system reaches a high level of complexity achieving sophisticated cognitive abilities. The sequencing of the genome of the *Octopus bimaculoides* revealed a striking expansion of TEs which were proposed to have contributed to the evolution of its complex nervous system. We recently found a similar expansion also in the genome of *Octopus vulgaris*. However, a specific search for the existence and the transcription of full-length transpositionally competent TEs has not been performed in this genus.

**Results:**

Here, we report the identification of LINE elements competent for retrotransposition in *Octopus vulgaris* and *Octopus bimaculoides* and show evidence suggesting that they might be transcribed and determine germline and somatic polymorphisms especially in the brain. Transcription and translation measured for one of these elements resulted in specific signals in neurons belonging to areas associated with behavioral plasticity. We also report the transcription of thousands of lncRNAs and the pervasive inclusion of TE fragments in the transcriptomes of both *Octopus* species, further testifying the crucial activity of TEs in the evolution of the octopus genomes.

**Conclusions:**

The neural transcriptome of the octopus shows the transcription of thousands of putative lncRNAs and of a full-length LINE element belonging to the RTE class. We speculate that a convergent evolutionary process involving retrotransposons activity in the brain has been important for the evolution of sophisticated cognitive abilities in this genus.

**Supplementary Information:**

The online version contains supplementary material available at 10.1186/s12915-022-01303-5.

## Background

Transposable elements (TEs) have contributed to the evolution of specific functions in a variety of biological systems and have given birth to a large fraction of vertebrate long non-coding RNAs (lncRNAs) [1–3]. Among TEs, retrotransposons move via a copy-and-paste mechanism using an RNA intermediate. The long interspersed element (LINE) L1, a non-LTR retrotransposon that predated the human genome, is active during neuronal differentiation [4] and causes somatic mosaicism establishing genomic variability in the brain [5]. Somatic L1 insertions are suggested to alter the transcriptional output of individual neurons, eventually affecting neuronal plasticity and behavior [6]. Transposition-driven genomic heterogeneity has also been documented in invertebrates including the neurons of mushroom bodies of *Drosophila melanogaster* [7] where they have been suggested to drive behavioral variability in individual flies. Negative regulators of retrotransposons are reported to be expressed in specific subgroups of neurons in *Aplysia californica* [8], *Drosophila melanogaster* [7], and in the mouse brain [9], further supporting the idea that, in nervous systems, retrotransposon transcription is finely regulated in a broad range of organisms and does not simply constitute noise. Our understanding of the activities of TEs in metazoa genomes is nevertheless still far to be complete, and the number of cellular events in which they have an influence is constantly growing. Novel findings allow us to increase our comprehension but also add layers of complexity to the topic. L1 elements, for example, have been demonstrated to have specific activity also at the non-coding level in the regulation of transcription and the organization of the genome [10, 11] and to be a component of extra-chromosomal DNA [12].

Among invertebrates, the cephalopod mollusk *Octopus vulgaris* is known for the richness of its behavioral repertoire achieving sophisticated vertebrate-like plasticity and neural control [13–16]. The remarkable complexity in the morphological and functional organization of its nervous system [14, 17, 18] is linked to evolutionary innovations at the cellular and molecular levels [17–21]. In the common octopus, about 500 million nerve cells constitute the nervous system, with about 300 million composing the nervous system in the arm and about 200 million nerve cells in what is considered to be the central brain [22]. This number results to be ten thousand times higher than that found in the sea hare *Aplysia* and still remains two hundred times higher when compared to the number of neurons present in the brain of the honeybee *Apis mellifera* (reviewed in [23]). This complexity at the cellular level is determined by key aspects of the transcriptional outputs of its genome such as large cadherin genes encoding over 70 extracellular cadherin domains; unprecedented expansions of gene families crucial for regulation, signaling, and cell communication (e.g., protocadherins, zinc finger proteins, G-protein coupled receptors); birth of many novel octopus-specific genes; the existence of a vascular endothelial growth factor (VEGF) pathway; reflectin genes originated by horizontal gene transfer; differential arrangements of key developmental genes; and extensive RNA editing capabilities [21, 24–28].

The sequencing of the *Octopus bimaculoides* genome [24] revealed an expansion of TEs and specific gene families related to transcriptional regulation and neuronal connectivity. Although no specific analysis was performed to identify full-length potentially active TEs and non-coding transcripts were discarded from the definition of the reference transcriptome, it was suggested that TEs are active in *O. bimaculoides* because a substantial fraction of RNAseq reads resulted in an overlap with TE fragments annotated in non-coding intergenic regions. Expansion of TEs has also been found in the genome of *Octopus minor* [29] and the other sequenced cephalopod species such as *Euprymna scolopes* [30] and the giant squid *Architeuthis dux* [31]. Performing a survey of the *O. vulgaris* genome, we have confirmed the expansion of TEs also in this species [32]. The current picture is that, in coleoid cephalopod species, repeated elements cover on average 45% of the genome while, in non-coleoid, this coverage is smaller reaching 30% in *N. pompilius* [33], 20% in *L. gigantea* [34], and 35% in *C. cigas* [35]. The TE expansion in coleoid cephalopod has been demonstrated to be due to retrotransposons [33].

Here, we sequence the *O. vulgaris* neural transcriptome to gain insights into the molecular composition underpinning its neural complexity. We identify a full-length LINE element and show that it is expressed especially in specific areas of the brain related to known forms of behavioral plasticity including learning. We also provide evidence for the transcription of thousands of long non-coding RNAs and the pervasive inclusion of TE fragments in coding and non-coding transcripts in both *O. vulgaris* and *O. bimaculoides*.

## Results

### Thousands of putative lncRNAs are expressed in the Octopus vulgaris nervous system

**Fig. 1:**
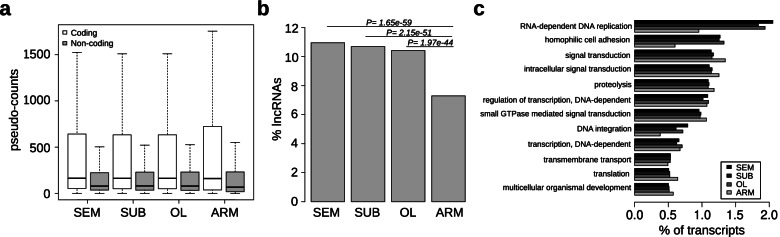
Features of the *Octopus vulgaris* brain and arm transcriptome. We sequenced the supra-esophageal (SEM) and sub-esophageal (SUB) masses and optic lobe (OL) as representatives of the brain and the medial segment of an arm (ARM), including the arm nerve cord, as the representative of the peripheral system. **a** Expression levels for coding and non-coding transcripts. Non-coding transcripts are on average expressed at lower levels than coding. **b** Percentage of the expressed non-coding transcripts. Brain sample results enriched for non-coding. **c** Percentages of transcripts expressed and their relative distribution among the most represented GO biological processes. A higher percentage of transcripts belonging to classes related to transposable elements and cell adhesion are expressed in the brain. Transcripts likely to be involved in signal transduction and translation constitute a larger quota in the arm

We generated a de novo assembly of the *Octopus vulgaris* neural transcriptome identifying and evaluating its functional annotations, potential lncRNAs, and repeats composition (Fig. 1; Additional file 1: Tables S1, S2, Figs. S1, S2, Additional files 2, 3 and 4). From each of three different octopus individuals, we collected four parts, three of them as representative of the central brain: (1) supra-esophageal mass (SEM), (2) sub-esophageal mass (SUB), and (3) optic lobe (OL), and one representing the peripheral nervous system: (4) a piece of the second left arm (ARM) including the arm nerve cord. We used these parts as a source of RNA for the sequencing (see the “Methods” section, Additional file 1: Table S1). The sequencing generated approximately 850 million paired-end reads accounting for 85 Gbp of sequence data that, following assembly and filtering, produced 64,477 unique transcripts (Additional file 2). The sequences showed a N50 value of 2087 bp, an average length of 1308, and about 38% average GC content (Additional file 1: Table S2). The transcriptome is more than 98% complete, and we functionally annotated 21,030 (32.6%) protein-coding transcripts (Additional file 1: Fig. S1 and Additional file 4). By performing stringent annotation analysis, a high proportion of transcripts (7806; 12.1%) was classified as putative lncRNAs (see the “Methods” section and Additional file 1: Fig. S2). In analogy to what is known about lncRNA expression in mammals [36], the non-coding portion of the *O. vulgaris* transcriptome shows a lower level of expression when compared to protein-coding genes (Fig. 1a) and a significantly higher number of lncRNAs is expressed in the central brain (~ 10%) with respect to the arm (~ 7%, *p*-value < 1e−40) (Fig. 1b). Functional enrichments highlight the differences between transcripts expressed in the central brain and in the periphery. Specifically, transcripts expressed in the brain are enriched in functions associated with neuronal cell adhesion and reverse transcription, while the ones expressed in the arm are enriched for functions associated with signal transduction and translation (Fig. 1c and Additional file 1: Fig. S3). We then mapped the transcriptome to the survey of the *O. vulgaris* genome [32]. Despite the quality of the current assembly being at the survey level and therefore rather fragmented, more than 34,000 transcripts were reliably mapped on the reference (Additional file ﻿5).

**Fig. 2:**
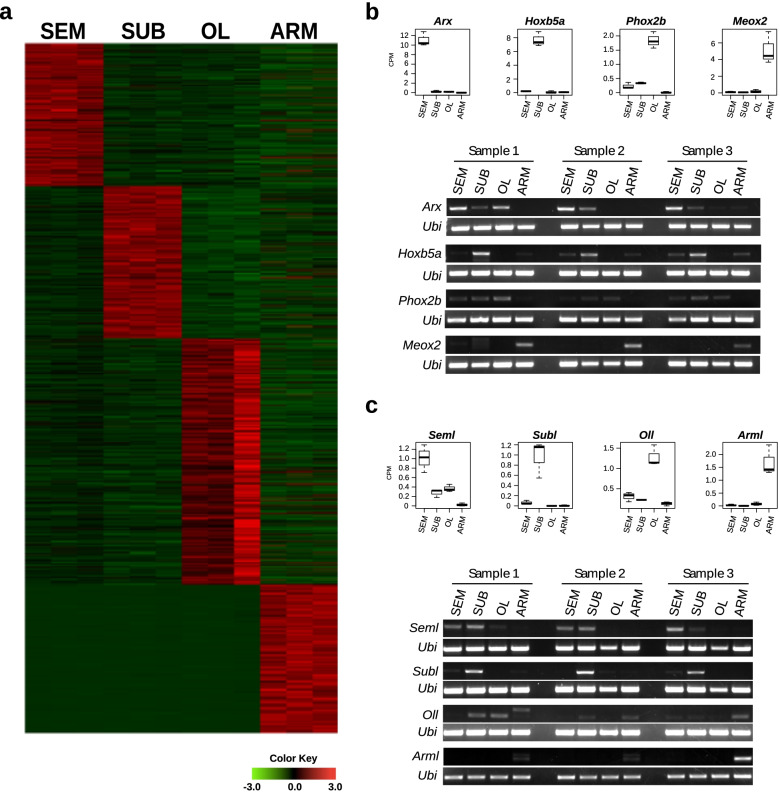
Transcripts with an expression peak and transcriptome validations. **a** Heatmap showing the expression levels for transcripts classified as having a peak of expression. **b** Boxplots showing the RNAseq expression levels of coding transcripts selected for validation and their relative RT-PCR results from 3 different individuals. **c** Boxplots showing the RNAseq expression levels of non-coding transcripts selected for validation and their relative RT-PCR results from 3 different individuals. The octopus ubiquitin transcript (Ubi) has been used as a positive control in all the experiments

We then validated the expression and the sequence of selected coding and non-coding transcripts by RT-PCR and Sanger sequencing (Fig. 2) selecting a group of transcripts showing a specific peak of expression in each of the collected parts. A transcript was considered to have a peak of expression in a given part when showing an expression level higher than 0.5 counts per million (CPM) in all three biological replicates of exclusively one part and below 0.5 in all the others. The value of 0.5 CPM was arbitrarily chosen as the value representing the 25 percentile of all the expression levels. This allowed the selection of ~ 1800 transcripts (~ 1500 coding and ~ 300 non-coding). Among the coding transcripts with an expression peak, only 54 resulted annotated. Among them, we noticed the presence of putative homologs of homeobox genes and selected 4 of them for validation through RT-PCR in 3 different individuals. The tested *Arx* putative homolog (Aristaless-related homeobox, comp31544_c0_seq1) is expressed mainly in the SEM by RNAseq and the RT-PCR validated this result. The RT-PCR also confirmed the peak of expression for the *Hoxb5a* putative homolog (homeobox B5, comp28131_c1_seq2) which is expressed mainly in the SUB and the *Meox2* putative homolog (mesenchyme homeobox 2, comp34840_c15_seq1) which is expressed mainly in the ARM. The *Phox2b* putative homolog (paired-like homeobox 2b, comp28142_c1_seq1) peaking in the OL by RNAseq data is expressed in all sampled parts of the brain by RT-PCR. For RT-PCRs concerning the lncRNAs, *Subl* (lncRNA with a peak of expression in the SUB, comp35227_c11_seq1) and *Arml* (lncRNA with a peak of expression in ARM, comp20195_c0_seq1) resulted to be tissue-specific, as they were mainly expressed in the SUB and in the ARM, respectively. On the other hand, *Seml* (lncRNAs with a peak of expression in SEM, comp18661_c0_seq1) showed to be expressed in the SEM but also in the SUB and the OL, while *Oll* (lncRNA with a peak of expression in the OL, comp35506_c7_seq1) presented expression in the OL but also in all the other sampled parts (Fig. 2c). Both *Oll* and *Arml* show the existence of two different isoforms in at least one individual. The RT-PCR results were generally in agreement with the sequencing data for both coding and non-coding transcripts tested. The identifiers indicated for every transcript are the same used in the Additional files and can be used to identify the corresponding sequences and annotations.

In order to verify the presence of lncRNAs also in *O. bimaculoides*, we reassembled the public RNAseq data from this species with the same method used for our *O. vulgaris* data (see the “Methods” section). We assembled 92,820 unique transcripts (Additional file 1: Table S2 and Additional file 6) of which 84,043 (90%) map on the published assembled genome with at least 90% coverage and 90% identity (Additional file 7). Our analysis demonstrated the presence, also in this octopus species, of thousands of putative lncRNAs (Additional file 8). Indeed, we were able to classify 10,030 assembled transcript as putative non-coding. They correspond to more than 10% of the assembled transcriptome of which 9132 map on the reference genome.

We then analyzed the conservation between the two octopus transcriptomes. Putative orthologs where identified by using BLAST and selecting for the reciprocal best hits (RBHs). From this analysis about 33,322 transcripts (50% of the assembled transcriptome) of the *O. vulgaris* transcriptome share orthologs with *O. bimaculoides*. The fraction of conserved transcripts is similar between coding (29,331, 52% of the coding transcripts with RBH) and noncoding transcripts (3991, 51%). In general, the average conservation of coding transcripts is slightly but significantly higher than that of lncRNA (93.3% vs 92.1%, *t*-test *p*-value = 1.1e−56). To evaluate the conservation of promoters, we extracted 1000 nucleotides upstream of the transcription start site of each transcript from each RBH pair that could be mapped on the genome in both species and contained enough nucleotides upstream of the transcription start site. Because the genomes are rather fragmented, about 18,000 RBH transcript pairs satisfied these conditions in both species and were considered in the analysis. On these pairs, we performed global alignments and classified as conserved those promoter pairs showing at least 50% identity. Randomization analysis building alignments between random promoter pairs showed the complete absence of pairs presenting such a level of conservation. From this analysis, 883 promoter pairs (5%) were classified as conserved between the two species. Interestingly, the proportion of conserved promoters from noncoding (7%, 171 out of 2451) is significantly higher (binomial test *p*-value 5.3e−07) with respect to promoters resulting conserved among promoter pairs of the coding transcripts (4.6%, 712 out of 15493). Finally, in order to test the conservation of the assembled transcripts at the positional level, we selected all the *O. vulgaris* contigs containing at least 10 mapped transcripts, which resulted in about 500 contigs, and identified the locations of the respective *O. bimaculoides* orthologs. On average, 85% of the ortholog transcript pairs are present on the same contig in the two species. We conclude that the transcripts are positionally conserved between the two species.

### A full-length LINE element is transcribed in the *Octopus vulgaris brain*

In order to evaluate the contribution of TEs to the *O. vulgaris* transcriptome, we analyzed its content in terms of repeated elements using RepeatMasker (Additional file 9) and found that more than 3.5 million nucleotides derive from interspersed repeats (4.5% of the total transcriptome content; Additional file 1: Table S3). More than 35% of the generated transcripts contain at least one interspersed repeat fragment. Among them, retroelements represent the TE fragments more frequently embedded in transcripts (26% of transcripts contain a fragment from at least one retroelement). According to the segregation of transposable elements in transcripts expressed in the different sequenced parts of the organism, we observed that SINE fragments are present in a higher fraction of transcripts expressed in the brain, while LINEs, LTRs, and DNA transposons are present in a higher portion of transcripts expressed in the periphery (Additional file 1: Fig. S4a). SINE also results in the class of retroelements more frequently embedded in lncRNAs (Additional file 1: Fig. S4b). We then asked whether the presence of head-to-head coding/noncoding pairs harboring the SINEUP [37] activity might be present in octopus. By screening the mapping of the transcriptomes generated in both species, we have found about 250 and 500 coding/noncoding overlapping pairs in *O. vulgaris* and *O. bimaculoides*, respectively. The 20% of these pairs contain a SINE element embedded in the lncRNA, but only few of them, 4 in *O. vulgaris* and 2 in *O. bimaculoides*, have a head-to-head orientation with the SINE fragment embedded in the non-overlapping part of the lncRNA resembling a canonical SINEUP element. We conclude that the SINEUP mechanism is probably not a frequent feature in the octopus genome.

**Fig. 3:**
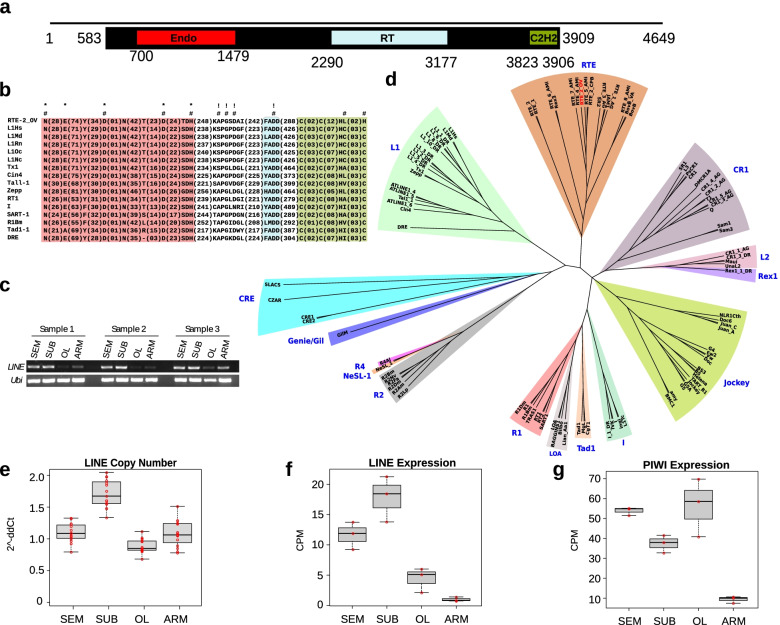
A full-length potentially active LINE transcribed in *Octopus vulgaris*. **a** Domain composition of the discovered LINE (comp36575_c1_seq3). The black line represents the transcript, the black box represents the location of the ORF, and the colored boxes represent the protein domains (Endo, endonuclease; RT, reverse transcriptase; C2H2, zinc finger). The numbers are relative to the nucleotide positions in the transcript. **b** Schematic alignment highlighting the conserved catalytic amino acids in the group of LINEs adapted from [38] plus the octopus element. The color code of the domains is the same as in **a**; amino acids critical for EN (*), RT (!), and retrotrasposition (#). **c** Electrophoresis from RT-PCR of the LINE showing the expression from three different animals. **d** Phylogenetic tree based on 100 LINEs from [39] (see Additional file 1: Table S4) plus the octopus element in red. **e** LINE copy number variation analysis using quantitative real-time PCR with Taqman probes. **f** Expression levels of the LINE based on RNAseq data. **g** Expression levels of Piwi-like protein 1 (comp33731_c0_seq1) from RNAseq data

To identify the molecular basis of the observed TEs expansion in *O. vulgaris*, we searched the transcriptome for putative autonomous active elements. We found a single element, a LINE mainly transcribed in the brain (Fig. 3a–c) that we named RTE-2_OV following phylogenetic analysis from which the element resulted to belonging to the RTE clade (Fig. 3d). The identified LINE presents an ORF of 3327 nucleotides and 5′ and 3′ UTRs of about 600 nucleotides. The translation corresponds to a 1109-amino acid polypeptide chain containing all the catalytic amino acids and domains needed for retrotransposition (Fig. 3a, b): a C-terminal endonuclease (EN), a reverse transcriptase (RT), and an N-terminal C2H2 zinc-finger (Znf) which is relatively rare in RTE elements [40]. It is unlikely that the strategy used to assemble the transcriptome might have assembled a full-length LINE with a complete ORF by putting together fragments from different independent transcriptional units. In order to rule out this possibility, we amplified the element from both gDNA and cDNA with a single PCR reaction. The longest single amplification goes from nucleotide 253 to 3903 of the assembled sequence. The fragment contains almost the entire ORF plus the majority of 5′ UTRs. There are only 6 nucleotides lacking at the end of the ORF because a microsatellite begins at the end of the ORF and made up the majority of the 3′ UTR. This did not allow a specific amplification of this region. Microsatellites are a common feature of RTE retrotransposons [41]. We cloned the obtained fragment from the cDNA amplification and validated it by primer walking and Sanger sequencing assembling a sequence with more than 98% identity to the reference. These results support the existence of the element in the genome and its transcription. The finding that this element contains an intact ORF and is transcribed indicates that it might be active and possibly drive retrotransposition. We then asked whether this element might show evidence of somatic retrotransposition. Southern blots analysis did not lead to conclusive results because of high background noise likely due to the high number of copies of this TE in the genome of *O. vulgaris*; however, the pattern observed is consistent with the existence of germinal and somatic genomic variations associated to the element (Additional file 1: Fig. S5). We also performed quantitative PCR experiments on DNA deriving from the four parts of different individuals. The results support the presence of different amounts of DNA from the element in different portions of the brain (Fig. 3e) showing an average higher content of RTE-2_OV DNA in the SUB with respect to the other parts. The existence of these increased amounts of RTE-2_OV DNA in SUB is in line with its higher expression in the SUB (Fig. 3f). Interestingly, the putative homolog of Piwi, known to repress the translation of TEs and therefore to restrict retrotransposition, displays a lower expression in the SUB with respect to the other portions of the central brain (Fig. 3g) consistently with what has been observed in the fruitfly brain [7].

### The RTE-2_OV element is mainly expressed in amacrine neurons

**Fig. 4:**
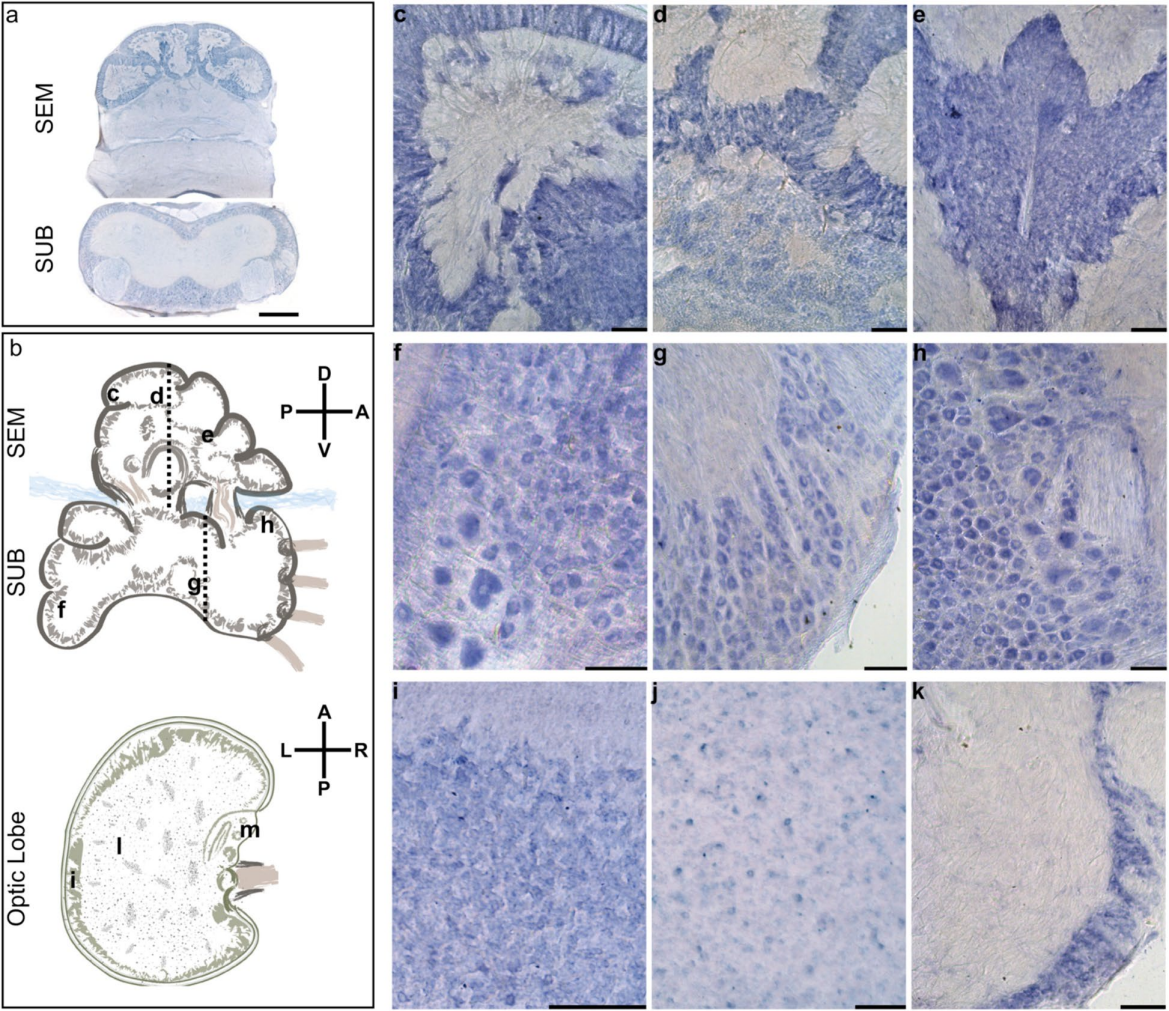
Localization of RTE-2_OV mRNA by in situ hybridization. **a** Bright-field micrographs of the coronal sections of the supraesophageal (SEM) and subesophageal (SUB) masses hybridized to digoxigenin-labeled LINE antisense (refer to the plane indicated by the dashed lines in **b**). The five gyri of the vertical lobe (dorsal in SEM) appear positively marked by the RTE-2_OV mRNA. Several cells in the cortical layers of the SUB appear also stained after in situ hybridization. Neuropils of SEM and SUB do not show a signal. **b** Schematic outline of the parts of the octopus brain for SEM and SUB (sagittal plane) and the optic lobe (OL; horizontal plane). Axes illustrating dorso-ventral and antero-posterior (SEM and SUB) and antero-posterior and left-right (OL) orientations with respect to the octopus body plan. Black letters indicate approximate levels of the sections provided in the other panels of the figure. **c** Detail of a gyrus of the vertical lobe (SEM) with densely packed amacrine cells showing a positive signal. **d** A similar signal in the gyri of the vertical lobe and some scattered positive cells in the sub-vertical lobe. **e** Section at the level of the sub-frontal lobe with densely packed amacrine small cells showing a strong positive signal. In the SUB, we observed **f** positive cells (20–25 μm in diameter) in the pallovisceral lobe and some larger neurons (40–50 μm) belonging to typical motor-center cellular types. **g** Cells (20–25 μm) belonging to the ventral side of the anterior pedal lobe and at the level of the dorsal brachial lobe (**h**) where some larger cells (up to 50 μm) are also marked after ISH. The small cells pertaining to these areas do not show positivity. Details of horizontal sections of the *O. vulgaris* optic lobe (**i**, **j**, **k**; in areas indicated in **b**): **i** Outer layer rich in intensely positive cells (small amacrine cells, < 5 μm), **j** inner medulla with scattered LINE mRNA-expressing cell bodies (up to 10 μm), and **k** cell bodies of the peduncle complex at the level of the median and posterior lobules of the olfactory lobe (cells of about 10 μm). Scale bars, 100 μm and 500 μm in **a**. Schematic drawings in **b** not to scale

To identify the domains of activity for the RTE-2_OV element, we performed RNA in situ hybridization and immunohistochemistry analysis. Localization of the RTE-2_OV transcript in *O. vulgaris* through in situ hybridization (ISH) showed specific expression of the element in subgroups of neurons in the brain and the absence of any signal in neuropils. We found most of the small cells of the sub-frontal lobe and of the five gyri of the vertical lobe as the most intensely stained areas in the supra-esophageal mass (SEM; Fig 4a–e; Additional file 1: Fig. S6b-e). Neural cells stained for RTE-2_OV create a tapestry closely matching the pattern obtained by DAPI (Additional file 1: Fig. S6b-c; Fig. S6d-e as control), indicating that the transcript is expressed in the great majority of the cellular bodies. We also found several positive neural cells in the sub-esophageal mass (SUB: anterior and posterior areas; Fig. 4f–h; Additional file 1: Fig. S6g) and in the optic lobe (OL; Fig. 4i–k; Additional file 1: Fig. S6l). In particular, after ISH, positive cells (20–25 μm in diameter) were observed at the level of the SUB in the pallovisceral lobe and some larger neurons (40–50 μm) belonging to typical cellular types of the motor-center present in the area. Positivity to the mRNA of the RTE-2_OV was also observed in cells belonging to the ventral side of the anterior pedal lobe (SUB) and in some larger cells (up to 50 μm) at the level of the dorsal brachial lobe (anterior part of the SUB). The small cells pertaining to these areas do not show any positive signal. In the optic lobe, the outer layer appeared rich in intensely positive cells (small amacrine cells, < 5 μm), and the inner medulla presented scattered cell bodies (up to 10 μm) expressing the element. Cell bodies of the peduncle complex at the level of the median and posterior lobules of the olfactory lobe also revealed a positive signal after ISH. We finally found isolated sparse large motor neurons positive at the level of the nerve cord in the arm (Additional file 1: Fig. S6s). No positive cells were observed in any muscle fiber in the arm or any other structure.

**Fig. 5:**
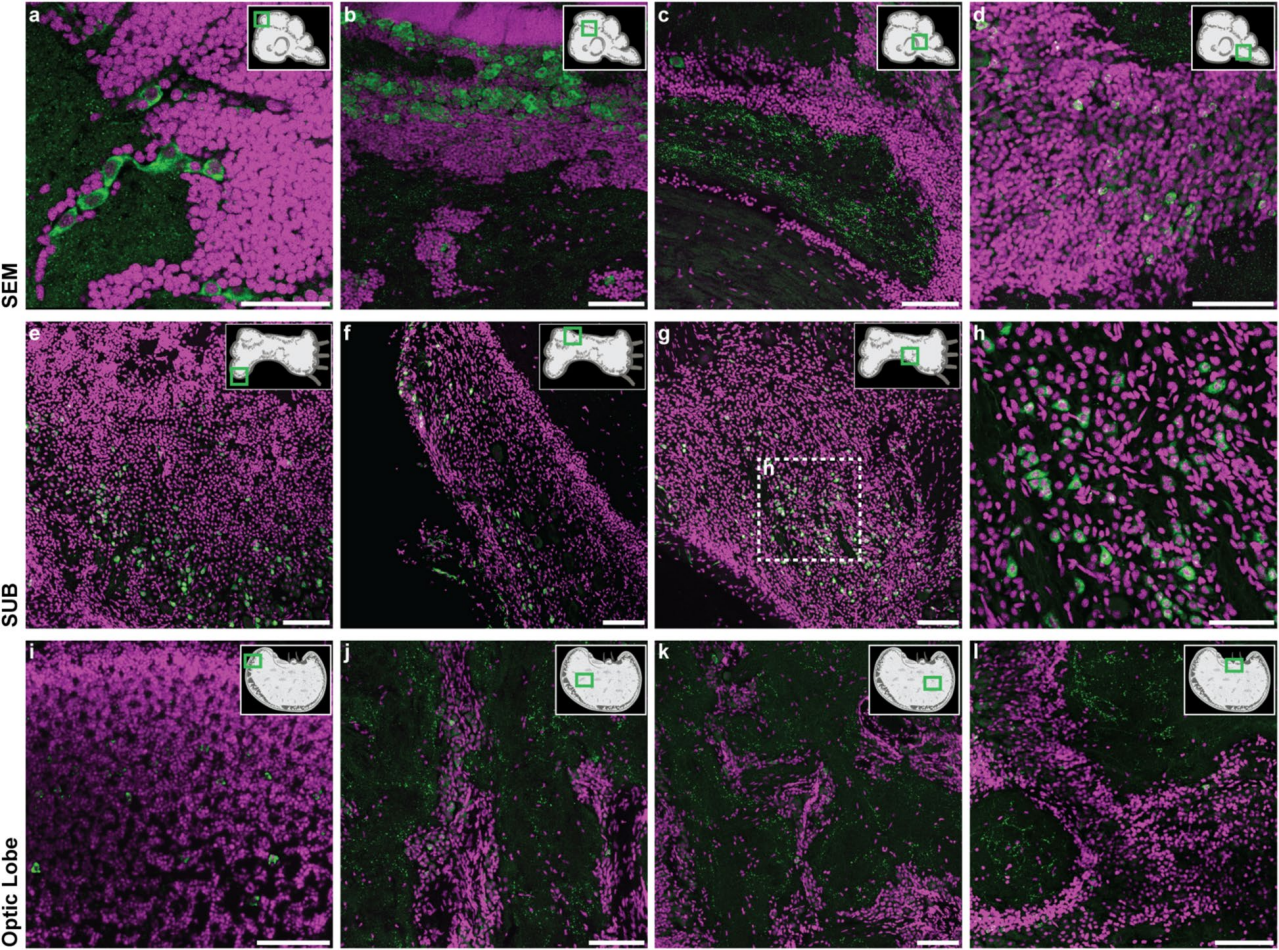
RTE-2_OV immunostaining in different areas of the brain. Coronal sections of the supra-esophageal (SEM; **a**–**d**) and sub-esophageal (SUB; **e**–**h**) masses and horizontal sections for the optic lobe (OL; **i**–**l**) following fluorescent-IHC (RTE-2_OV signal in green, DAPI used as a nuclear stain in magenta) highlight a differential pattern of positive cells and fibers in *O. vulgaris* brain. A schematic drawing of the brain parts is provided with areas of interest indicated in the green square. **a** Large positive cells are found in the vertical lobe (VL). These appear organized in trunks and clearly distinguishable from the population of numerous amacrine cells constituting the VL (DAPI stained layer). **b** Large cells in the sub-vertical lobe (cellular wall) and a part of the bundle of fibers are present at the beginning of the sub-frontal lobe (**c**). **d** Scattered positive cells are also identified in the posterior buccal lobe. Several positive cells are identified in the SUB in the cellular walls of the vasomotor lobe (**e**) and in discrete areas of the pedal lobe (**f**). A similar pattern of positive cells is recognized at the level of the anterior part of the pedal lobe (**g**). A detail in the higher magnification (**h**; square in **g**) of the cellular layer of the lobe serves to highlight the population of positive cells. In the OL, several amacrine cells are found positive in the external granular layer (**i**). The OL-medulla is populated by few immune-reactive neurons found in the cellular islands (**j**, **k**), and positive fibers dispersed in the surrounding neuropil (**j**, **k**). Positive cells and fibers are also identified in the peduncle lobe. The internal layer of cells of the neural wall of the olfactory lobe and the spine (**l**) are shown. Scale bars, 100 μm, with the exception of **a** and **h** (50 μm)

Immunohistochemistry (IHC) with RTE-2_OV custom antibodies identified in the vertical lobe a number of large cells organized in trunks and positive fibers in the neuropil of the gyri. We also observed large positive cells organized in the chain at the level of the cellular wall of the sub-vertical lobe (Fig. 5b) and few positive neurons in the great majority of the islands of cells in the posterior wall of the sub-vertical lobe. A distinct pattern of positive fibers was found in the neuropils of the SEM (Fig. 5a–c) together with a scattered number of cell bodies (see areas belonging to superior and inferior frontal lobes; Fig. 5c, d). We did not identify positive fibers in the SUB, but only a distinct population of a small number of neurons in the vasomotor lobes (Fig. 5e) and in discrete areas of the anterior and the lateral posterior wall of the pedal lobe (Fig. 5f–h). Immuno-reactivity was also evident in several scattered amacrine cells of the external granular layer of the OL (Fig. 5i). We found several RTE-2_OV-positive fibers dispersed in discrete areas of the neuropil of the OL and a distinct pattern of positive cells and fibers in the peduncle lobe, mostly toward the internal layer of cells of the neural wall of the olfactory lobe and the spine (Fig. 5l). A small number of large cells dispersed in the arm nerve cord were also found to be stained (data not shown).

### Identification of a potentially active LINE in *Octopus bimaculoides*

**Fig. 6:**
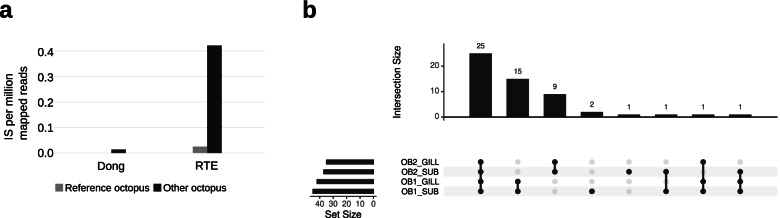
Identification of a putatively active LINE transcribed in *O. bimaculoides*. **a** Normalized number of non-reference insertions in two different *O. bimaculoides* individuals (octopus used for the reference genome and a different octopus individual) for the two LINEs identified in this species. **b** Upset plot showing the non-reference insertions identified in the 30X WGS from two different samples from 2 different individuals and how they are shared between the four samples. OB1_SUB: sample from the subesophageal mass of the octopus 1. OB1_GILL, sample from the gill of octopus 1; OB2_SUB, sample from the subesophageal mass of the octopus 2; OB2_GILL, sample from the gill of octopus 2

In order to add further support to the idea that LINE elements might be active in the genomes of the *Octopus* genus, we searched the public genome, transcriptome, and our custom transcriptome assembly (Additional files 6, 7 and 8) of *O. bimaculoides* for the presence of assembled full-length LINEs. We were not able to identify any assembled full-length LINE in the transcriptome nor in the genome. However, when inspecting the repeat library consensus generated in the work by Albertin et al. [24], we identified two LINEs with a full-length ORF and the complete set of domains: an RTE and a Dong [42] element. To gain insights into the possibility that the identified LINE elements might be active, we generated DNAseq sequencing data from the optical lobe of an *O. bimaculoides* individual and searched for novel integration sites (IS) with respect to the reference genome using MELT [43]. The same analysis was performed using the public DNAseq data derived by the gonads of the individual used to assemble the reference *O. bimaculoides* genome. Comparing the whole-genome sequencing data from the two different *O. bimaculoides* individuals, we obtained significant evidences of activity only for the RTE element. Indeed, the element showed a significantly higher number of non-reference ISs with respect to the reference individual (Fig. 6a, binomial test *p*-value = 4.8e−69) which indicates that the element is at the basis of polymorphisms between the two individuals. Conversely, the Dong element resulted in a very low number of non-reference ISs which does not significantly differ from the results obtained with reads from the reference individual and therefore does not display evidences of recent activity. In order to further validate the potential activity of the element discovered and evaluate the existence of somatic events, we sequenced at an average depth of 30× per sample the DNA from two different tissues from two different *O. bimaculoides* individuals. We selected a neural tissue (SUB) and a non-neural tissue (GILL). MELT split was used to identify non-reference insertion sites of the RTE element (Additional file 10). Following filtering, we identified 55 ISs, 25 of them are present in all the samples and represent polymorfisms common to both individuals; 15 are specific of one individual while 9 are specific of the other (Fig. 6b). In addition, 3 ISs appear to be specific of the SUB of only one of the two individuals and therefore represent potentially somatic events. Finally, the remaining 3 ISs are inconsistently identified in both the individuals but not in both tissues. The presence of a substantial number of elements present in both tissues and individuals and those individual-specific add evidences to the idea that this element might be active and that our analysis is robust. On the other hand, these results suggest that the retrotranspositional activity of the element at the somatic level is probably limited. Although we cannot completely rule out the existence of somatic activity at the very cell-specific level, the small number of putative somatic insertions identified depose against a pervasive level of somatic retrotransposition mediated by this element. Further studies taking advantage of single-cell whole-genome and long-reads sequencing will definitely answer the question whether the activity of such elements is capable of generating unique polymorphisms in the genome of every single cell of the brain and if this has any functional outcome.

## Discussion

The analysis of the coding part of the *O. bimaculoides* genome provided interesting parallelisms with the transcriptional output of mammalian genomes [24]. Our results in *O. vulgaris* support and expand this view suggesting that the molecular organization of the octopus neural transcriptome resembles the mammalian one also for what concerns the transcription of thousands of lncRNAs, their TEs content, and transcription of retrotransposons.

Here, we report the assembly of the most complete transcriptome for the *Octopus vulgaris*. The sequencing of different portions of the organism allowed us to identify transcripts whose expression showed a peak in only one part. For PCR validations, we chose putative homologs of homeobox genes. *Arx* is a homeobox gene associated with mental retardation. In mammalian, it has been shown to be expressed in the fetal brain at various developmental stages, mainly in neuronal precursors in the telencephalon where it results involved in corticogenesis and in the differentiation and maintenance of specific neuronal cell types [44, 45]. In our *O. vulgaris* transcriptome assembly, its putative homolog results to be expressed mainly in the SEM consistently with an important function in the central brain also in this species. As another example, the expression of the putative homolog of *Meox2* appears to be restricted to the arm in *O. vulgaris*, which also appears consistent with the known function of this gene in vertebrates where it controls limb muscle development [46]. The selection and validation of these expression peaking transcripts, together with the functional information of the putative homologs, added support to the quality of the assembled transcriptome and allowed us to perform more specific analyses. We observed that more than 10% of the assembled transcriptome for *O. vulgaris* and *O. bimaculoides* is composed of putative lncRNAs and that more than 35% of the assembled transcripts contain at least one fragment derived from a transposable element. Despite the fact that different classes of TEs appear to be differentially segregated between coding and non-coding transcripts, we did not find an overall difference nor a strong enrichment for lncRNAs to contain embedded TE fragments with respect to coding ones. This result was unexpected and warrants further investigation. One of the causes might rely on our conservative strategy to call non-coding transcripts for which we might likely have underestimated the fraction of lncRNAs increasing the rate of false negative. Another reason for a possible underestimation of the fraction of lncRNAs relies on the fact that they present a generally lower expression level with respect to protein-coding genes in bulk RNA samples. This is often due to the fact that they are expressed in a limited and specific number of cells. Single-cell approaches are an important emerging technique that will help to explore this and similar issues.

The observation reported from us and others about TE expansion in octopus and generally in coleoid cephalopods with respect to non-coleoid cephalopods and sister species drove our interest toward the proportion of the transcriptome deriving from transposable elements. This lead us to the discovery of a novel RTE LINE retrotransposon transcribed in the central nervous system of *O. vulgaris*. Reanalysis of the *O. bimaculoides* data allowed us to identify, also in this species, a LINE member of the RTE clade showing evidences of recent activity. We could not find any full-length copy of the *O. vulgaris* element in the genome survey [32] nor could we find the *O. bimaculoides* element full-length sequence in its reference assembly. This likely resulted from the difficulties to assemble a reference genome using exclusively short reads in regions containing long repeated sequences as those containing full-length LINE elements. Despite this technical gap, the complementary use of transcriptomic, repeats reconstruction, annotation data, and manual curation allowed us to identify the full-length autonomous elements likely to be active in both the octopus species. In addition, we were able to amplify from gDNA and cDNA the *O. vulgaris* element and to clone and validate by Sanger sequencing the amplified fragment obtained by the cDNA, therefore confirming the assembled element sequence and its transcription. The identification of potential integration polymorphisms of the RTE element in *O. bimaculoides* also adds support to its putative activity. Also, the results from quantitative PCR and Southern blot analysis relative to the element discovered in *O. vulgaris*, although not conclusive, support the existence of somatic retrotransposition in the central brain for this species. It is here important to consider that results from quantitative PCR for LINE genomic copy number variations should be interpreted with care, especially in case of the absence of additional supporting evidences. Indeed, in addition to a potential variation in genomic copy number, they might also reflect a different amount of extra-chromosomal DNA which, at least in humans, is composed of a consistent fraction of LINE DNA [12, 47]. Our WGS of two different tissues from two different *O. bimaculoides* individuals identified very few potential somatic insertions and does not corroborate the idea of pervasive somatic retrotransposition. More specific and careful inspections are needed to validate, beyond any reasonable doubt, the activity of the elements presented in this work. Nevertheless, our results represent the first evidences of LINE activity conserved in the *Octopus* genus.

Through ISH, we localized RTE-2_OV transcripts in specific groups of neural cells and areas of the *O. vulgaris* nervous system, with no signal in neuropils. In particular, positive signals were observed at the level of the majority of amacrine cells (about 3 μm diameter) which are suggested to have very limited neural processes [48]. The most intriguing signals are those present at the level of the sub-frontal lobe and of the five gyri of the vertical lobe (VL). These areas appeared to be the most intensely positive to RTE-2_OV transcript found in the supra-esophageal mass. VL represents about 14% of the overall volume of the SEM in the adult *O. vulgaris* [49] and counts about 25 million neural cells, equivalent to more than 65% of the total number of nerve cells estimated for the SEM in the common octopus [22]. The largest quota of this impressive number of closely packed nerve cells is represented by the amacrine cells, the smallest in the octopus brain. Lying below the vertical lobe is the sub-vertical lobe. Its neural architecture is characterized by the presence of a wall that in several regions is folded to form islands of cells. Many cells have a diameter less than 5 μm and very few are larger than 10 μm. At both sides of the sub-vertical lobe, there are cells with a diameter of 5–10 μm, but also the largest neurons of the whole SEM reaching 25 μm. Through IHC in these areas, we identified a number of large cells positive to RTE-2_OV antibodies appearing organized in trunks as typically described by Young in 1971 [50]. We also observed large positive cells organized in chains at the level of the cellular wall of the sub-vertical lobe, and few positive neurons in the great majority of the islands of cells (posterior wall of the sub-vertical lobe). Finally, immuno-reactivity was also evident in several scattered amacrine cells belonging to the external granular layer of the OL suggested to correspond to the neural cells described to project in the underlying plexiform zone [15].

Remarkably, *O. vulgaris* posterior frontal and vertical lobes belong to a neural circuit considered to be functionally analogous to the mammalian hippocampus and limbic lobe [15, 17, 51, 52] where retrotransposons are also reported to be active [7, 53]. The VL is also suggested to be analogous to the mushroom bodies of insects [54]. All other octopus brain areas where the mRNA and/or the protein were identified are centers of active neural plasticity for visual sensory-motor processing and memory (SEM and OL), and motor processing (SUB) [17, 50, 55]. The positive cells overlap with the intricate neural network made by amacrine cells known to act as amplifying matrices functioning as read-in read-out memory of both sensory-motor visual and chemo-tactile processing [15, 52, 56, 57].

The reported imaging experiments allow also to made observations at the cellular level. The signals resulting from IHC and ISH are principally cytoplasmic. A higher number of cells appear to contain the element mRNAs with respect to the cells in which the protein can be identified. We only observed a partial overlap between cells expressing the mRNA and cells containing the protein. These observations might reflect the complexity of LINE involvement in cellular activities. Such activities might be resulting by the strategies employed in the cell to contrast retrotransposition, but they can also reflect the basis of specific LINE functions that we currently understand only with limitations. Most of our knowledge of LINE elements comes from studies on human L1. Its transcript is a bicistronic mRNA which codifies for two open reading frames (ORF1 and ORF2). Among them, the ORF2 protein contains the endonuclease, reverse transcriptase, and zinc finger domains, and therefore, it can be considered the ORF codifying for the retrotranspositional machinery and the functional homolog of the single ORF of the RTE elements that we found in the two octopus species. Of note, the two human L1 ORFs do not result to be translated at the same levels. ORF1 is translated at a higher level than ORF2, and different proteins which repress ORF2 translation have recently been identified [58]. Consequently, the ORF2 protein has been observed to be absent in a portion of cells where the mRNA and ORF1 protein were present [59]. The limited efficiency for the translation of ORF2 has been proposed to be one of the mechanisms that the cell employs to limit and/or regulate the retrotranspositional potential of the L1 mRNAs. Indeed, for the retrotransposition to take place, all the L1 components, ORF1 protein, ORF2 protein, and L1 mRNA must be present in the same ribonucleoprotein complex (RBP) within the nucleus. The lack of any of these components in the RBP does not allow retrotransposition. Reasoning along this line, another mechanism used to restrict retrotransposition is the confinement of the RBP complex within the cytoplasm, where it is assembled. As long as the complex is located in the cytoplasm, it cannot access the genome and therefore cannot retrotranspose. For this reason, cells subjected to retrotransposition have been identified mainly in cells which underwent duplication. The mechanism proposed is that the RBP can get in contact with nuclear DNA only after the nuclear membrane dissolves during mitosis. If the RBP remains in contact with the DNA, it is then found in the nucleus after the nuclear membrane of the new cell has been rebuilt thus allowing retrotransposition [59, 60].

Spatial uncoupling between L1 mRNA and proteins can also be observed independently from cellular defense mechanisms. Indeed, L1 elements are also active at the mRNA level as lncRNAs. During early development and in the regulation of cell potency, L1 transcription and its mRNA result to play a crucial role in regulating global chromatin accessibility and the L1 mRNA can act as an RNA scaffold to recruit specific regulatory proteins to the genome [10, 11]. Another molecular event resulting in spatial uncoupling of LINEs transcription/translation can be observed when LINE mRNA is used from a given cell type to target a different cell type with retrotranspositional activity. In fruitflies, the existence of a mechanism producing mRNAs of the I element (another member of the LINE class) by the nurse cells has recently been observed. Once produced, these mRNAs are transported to the transcriptionally inactive oocytes where they are translated and can cause retrotransposition (Wang et al. [61]). While it is still debated whether retrotranspositional activity has a specific role during development and differentiation and in the functioning of the brain, it has been consistently reported that mRNAs from retrotransposons are specifically needed for chromatin organization and transcriptional regulation during development and differentiation especially in the brain [10, 11, 62–65]. This raises the question whether somatic retrotransposition has a functional meaning in the brain of metazoa or if it is simply a byproduct of the retrotransposons function at the transcript level.

## Conclusions

Our data corroborate the hypothesis that LINE elements might be active and functionally important in the central nervous system of highly intelligent organisms such as octopuses [6]. The localization of RTE-2_OV mRNA into different neural cells and not in the neuropil or muscle, complemented by the distinctive pattern of expression of its protein product and the identification of a potentially active element of the same class from a congeneric species, support the view that RTE-2_OV might be functional in neural centers in *O. vulgaris* and make it an important candidate to be further studied for its contribution to neural plasticity in this fascinating organism.

## Methods

### Animals, sampling, and ethical statement

*Octopus vulgaris* were collected by local fishermen from the Bay of Naples (Southern Tyrrhenian Sea, Italy) in early summer 2012. Animals were transported to the Stazione Zoologica Anton Dohrn in Napoli and maintained according to a standardized acclimatization protocol [66–68]. Samples were taken from local fishermen, by applying humane killing following principles detailed in Annex IV of Directive 2010/63/EU as described in the Guidelines on the Care and Welfare of Cephalopods [68] and following protocol for collection of tissues described by Baldascino and coworkers [69]. Death was confirmed by transection of the dorsal aorta. All dissections were carried out on a seawater ice bed. During surgery optic lobes (OL), supra- (SEM) and sub-esophageal (SUB) masses were dissected out from the animal, a piece of an arm (ARM), usually the second left arm, was also taken. The complete dissection lasted less than 10 min. Sampling from live animals occurred before the entry into force of the Directive 2010/63/EU in the Member States, and therefore, no legislation was in place in Italy regulating research involving cephalopods. However, the care and welfare of animals have been consistent with best practice [68, 70, 71] and in compliance with the requirements of the Directive 2010/63/EU that includes cephalopods within the list of species regulated for scientific research involving living animals. In addition, animals killed solely for tissue removal do not require authorization from the National Competent Authority under Directive 2010/63/EU and its transposition into national legislation.

### RNA extraction and sequencing

For each octopus (*N* = 3), total RNA was isolated from central nervous tissues (SEM, SUB, and OL) and ARM, a part of the body including the largest quota of the neuronal population belonging to the peripheral nervous system [22, 72] and thus constituted by muscle and peripheral nervous tissue. SV total RNA isolation kit (Promega, #Z3100) was utilized according to the manufacturer’s protocol. DNA was degraded by treating samples with Turbo DNase Kit (Ambion) according to the manual. The quality and quantity of RNA were assessed by NanoDrop (Thermo Fisher) and RNA BioAnalyzer (Agilent Technologies, Santa Clara, CA, USA). Paired-end libraries were prepared using the Illumina TruSeq RNA sample library preparation kit (Illumina, San Diego, CA, USA). Each sample was barcoded, and samples pooled and sequenced in two lanes on the Illumina HiSeq 2000 platform (paired-end, non-strand specific, 2 × 50 bp read length protocol).

### Raw reads quality filtering

Quality of raw reads was assessed using FastQC (release 0.10.1). Raw reads were filtered and trimmed based on quality and adapter inclusion using Trimmomatic [73] (release 0.22; parameters: -threads 24, -phred 64, ILLUMINACLIP:illumina_adapters.fa:2:40:15, LEADING:3, TRAILING:3, SLIDINGWINDOW:3:20, MINLEN:25). Read pairs with both reads passing the filters were considered for the transcriptome assembly. Trimmed and filtered reads were normalized to remove duplicates using the normalize_by_kmer_coverage.pl script from Trinity [74] (release r2013_08_14; parameters: --seqType fq, --JM 240G, --max_cov 30, --JELLY_CPU 24).

### De novo assembly and quantification of transcript abundances

Transcriptome was assembled using Trinity (release r2013_08_14) on the trimmed, filtered, and normalized reads exploiting the Jaccard clip to limit assembly of chimeras. Assembly was performed using the following parameters: --seqType fq, --JM 240G, --inchworm_cpu 24, --bflyHeapSpaceInit 24G, --bflyHeapSpaceMax 240G, --bflyCalculateCPU, --CPU 24, --jaccard_clip, --min_kmer_cov 2. To measure expression levels, raw reads were mapped on the assembled transcriptome using Bowtie (version 1; parameters: -p 24, --chunkmbs 10240, --maxins 500, --trim5 2, --trim3 2, --seedlen 15, --tryhard -a -S). SAM outputs from Bowtie [75] were converted into BAM, sorted, indexed, and counted using the view, sort, index, and idxstats programs from Samtools [76]. All transcripts not showing at least 0.5 reads mapping per million mapped reads (CPM) in at least 2 samples were discarded from the transcriptome as being expressed at too low levels and therefore likely deriving by noise or assembly artifacts.

### Annotation and mapping of the assembled transcriptome

CEGMA (Core Eukaryotic Genes Mapping Approach; release 2.5) [77] was used to measure the completeness of the assembled transcriptomes using the set of 248 Core Eukaryotic Genes (CEGs). Transcripts annotation was performed using the Annocript pipeline [78] (release 0.2) with the combination of tool, parameters, and databases described below and using BLAST+ (release 2.2.27) [79]. To annotate proteins, we used BLASTX against the UniRef90 and Swiss-Prot databases from UniProt (release 2013_09) [80] with the following parameters: -word_size 4, -evalue 10e-5, -num_descriptions 5, -num_alignments 5, -threshold 18. To annotate protein domains, we used RPSBLAST against the Conserved Domains Database (CDD v3.10) [81] with the following parameters: -evalue 10e-5, -num_descriptions 20, -num_alignments 20). Ribosomal and small non-coding RNAs were identified using BLASTN against a custom database made by Rfam (realease 11.0) [82] and ribosomal RNA sequences from GenBank (parameters: -evalue = 10e-5, -num_descriptions 1, -num_alignments 1). Each transcript was associated to Gene Ontology (GO) [83], Enzyme Commission (EC) [84], and UniPathway [85] through cross-mapping of the best match from UniRef90 or Swiss-Prot using the annotation mapping tables from UniProt. For each transcript, we used the Virtual Ribosome (Dna2pep release 1.1) [86] to predict the length of the longest ORF searching across all reading frames without the constraint to begin translation from a methionine start codon (parameters: -o none, -r all). The non-coding potential for each transcript was calculated using Portrait (release 1.1) [87]. The *Octopus vulgaris* reference genome survey was downloaded on March 2021 from https://springernature.figshare.com/ndownloader/files/13876385. Assembled and filtered unique transcripts were mapped on the genome using gmap [88] (parameters: --suboptimal-score 0 -f gff3_gene --gff3-add-separators 0 -t 32 --min-trimmed-coverage 0.9 --min-identity 0.9) considering only transcripts aligning at least 90% of their length with 90% minimum identity. We were able to map 34,239 (~ 53%) transcripts.

### Non-coding annotation of the assembled transcriptome

Putative lncRNAs were classified based on a heuristic process considering the annotation results. The constraints used to identify potential lncRNAs have to be considered very stringent (Additional file 1: Fig. S2). In published studies, different combinations of analyses have been used to identify lncRNAs [87, 89–91] (1) lack of similarity with proteins, (2) lack of similarity with domain profiles, (3) lack of similarity with other RNAs (ribosomal, snoRNA, etc.), (4) transcript and longest ORF lengths, and (5) non-coding potential. We put all these together and classified as lncRNA only those transcripts satisfying all the following conditions: (a) length ≥ 200 nucleotides; (b) lack of similarity with any of the following: protein from Swiss-Prot and UniRef90, domains from CDD, rRNA from GenBank, and other small ncRNA from Rfam; (c) longest ORF < 100 amino acids; and (d) non-coding potential score ≥ 0.95. Using these stringent constraints, we were able to predict in the *O. vulgaris* transcriptome 7806 (~ 12%) transcripts as putative lncRNAs.

### Assembly, mapping, and annotation of the *Octopus bimaculoides* public RNAseq data

*O. bimaculoides* RNAseq raw data from Albertin et al. [24] were downloaded from NCBI SRA in October 2015 using the SRA Toolkit. Raw reads were filtered and trimmed based on quality and adapter inclusion using Trimmomatic (release 0.33; parameters: -threads 32, ILLUMINACLIP:illumina_adapters.fa:2:40:15:10:true LEADING:3 TRAILING:3 SLIDINGWINDOW:3:20 MINLEN:50). Read pairs with both reads passing the filters were considered for the assembly. Trimmed and filtered reads were assembled with Trinity (release 2.1.0; parameters: --seqType fq --SS_lib_type RF --CPU 32 --max_memory 240G --inchworm_cpu 32 --bflyHeapSpaceInit 24G --bflyHeapSpaceMax 240G --bflyCalculateCPU --normalize_reads --min_kmer_cov 2 --jaccard_clip) using digital normalization, strand information, the Jaccard clip and assuring that every kmer used in the assembly was present in at least 2 reads to reduce noise. Redundancy of assembled transcripts was reduced using Cd-hit [92] (version: 4.6, parameters: -c 0.90 -n 8 -r 0 -M 0 -T 0). To measure the expression levels, raw reads were mapped on the transcriptome using Bowtie (version 1, parameters: -t -q -p 32 --chunkmbs 10240 --maxins 500 --trim5 2 --trim3 2 --seedlen 28 --tryhard -a -S). SAM outputs from Bowtie were converted into BAM, sorted, indexed, and counted using the view, sort, index, and idxstats programs, respectively, from the Samtools software collection. All transcripts not showing at least 1 reads mapping per million mapped reads (CPM) in at least 1 sample were discarded from the transcriptome. *Octopus bimaculoides* genome was downloaded on August 2015 from http://genome.jgi.doe.gov/pages/dynamicOrganismDownload.jsf?organism=Metazome.

Assembled and filtered unique transcripts were mapped on the genome using gmap [93] (version: 2015-09-28, parameters: --suboptimal-score 0 -f gff3_gene --gff3-add-separators 0 -t 32 --min-trimmed-coverage 0.9 --min-identity 0.9) considering only transcripts aligning at least 90% of their length with 90% minimum identity. We were able to map 84,043 (~ 90%) transcripts. Annotations and all the remaining analysis were executed as for *O. vulgaris*.

### Conservation analysis and SINEUP search

Putative orthologs between *O. vulgaris* and *O. bimaculoides* species were identified using BLAST+ (program blastn, parameters: -best_hit_overhang 0.1, -evalue 1e-0.5) and searching for reciprocal best hit (RBH). Promoters were defined as 1000 nucleotides upstream the annotated transcription start site and extracted for each ortholog pairs for which both transcripts could be mapped on the respective genome presenting enough sequence space upstream the transcriptional start site. Promoter pairs were aligned among them by using the function pairwiseAlignment from the Biostrings Bioconductor [88] package in R using default parameters. To identify positional conservation between the two species, we selected all the *O. vulgaris* scaffolds containing at least 10 mapped transcripts and counted how many pairs of orthologs were present on the same scaffolds in both the species. To search potential SINEUP, in each species, we used the GenomicRanges Bioconductor package [94]. Basically, we collected the closest transcripts for each pair (mRNA/mRNA, lncRNAs/lncRNAs, mRNA/lncRNAs). We then parsed the RepeatMasker output calculating the coverage of the repeats for each transcript and selected the mRNA/lncRNAs pairs with head-to-head overlap having at least one SINE element in the non-overlapping part of the lncRNA.

### Identification and classification of repetitive elements

Repetitive elements for each transcriptome were annotated using RepeatMasker (A.F.A. Smit, R. Hubley & P. Green RepeatMasker at http://repeatmasker.org; release 4.0.5) searching against the Repbase database [95] (release 20140131) with parameters: -species bilateria, -s, -gff. We counted from RepeatMasker output the repeat fragments present at least once in each transcript and built a matrix containing the percentage of transcripts containing fragments related to (a) retroelements, (b) DNA transposons, (c) satellites, (d) simple-repeats, (e) low complexity, (f) others, and (g) unknown classes for each transcriptome according to the RepeatMasker classification.

### Identification of full-length transposable elements

We parsed RepeatMasker output calculating the percentage of overlap between the assembled transcripts and the repeat consensus from Repbase selecting all elements with at least 80% coverage on the repeat consensus. Elements showing the highest coverage were selected. On these, we used Virtual Ribosome to predict the longest complete ORFs by searching across all reading frames with methionine as start codon and a canonical stop (parameters: -o strict, -r all). A single transcript resulted with a complete ORF. On this, we used InterPro [96] to identify and classify protein domains. The potential catalytic amino acids essential for the retrotransposition were manually identified comparing the putative translation with those reported in Clements and Singer [38]. The same analysis was performed on both the transcriptomes of *O. bimaculoides* (assembled by Albertin and assembled by us). The analysis was also performed on the RepeatMasker annotation of the genome by Albertin downloaded from http://octopus.unit.oist.jp/OCTDATA/. For consistency, we also analyzed RepeatMasker annotations of the genome and the transcriptomes produced using the same tool, library, and parameters used for *O. vulgaris* and the other species considered in this study. In no one of the analyses, we could find a full-length transposable element retaining a complete ORF for *O. bimaculoides*. We then translated the main RepeatScout and RepeatModeler repeat libraries consensuses assembled by Albertin et al. (main RepeatScout library: http://octopus.unit.oist.jp/OCTDATA/TE_FILES/mainrepeatlib.fa.gz; RepeatModeler library: http://octopus.unit.oist.jp/OCTDATA/TE_FILES/oct.rm.tar.gz) with the Virtual Ribosome tool to predict longest ORF searching across all reading frames showing methionine as start codon (parameters: -o strict, -r all) and a canonical stop. The InterPro tool was then used to identify and classify the LINE characteristic domains. The potential catalytic amino acids essential for the retrotransposon activity were manually identified by comparing the ORF sequences with those reported in Clements and Singer. This led us to the identification of two potentially functional LINE retrotransposons.

### Identification of a potentially active retrotransposon in *O. bimaculoides*

The two candidate retrotransposons found in *O. bimaculoides* RepeatModeler libraries were analyzed to search for integration sites in gonads and optic lobe using MELT [43] (v2.0.2). Two genomic DNAseq WGS libraries from gonads (SRR2010220 and SRR2005790) were downloaded from the European Nucleotide Archive (ENA) at https://www.ebi.ac.uk/ena/data/view/PRJNA270931. We generated two additional DNAseq WGS libraries from DNA extracted from the optic lobe (L001 and L002) of a different individual. *O. bimaculoides* reference genome was filtered for scaffold shorter than 10,000 bp and reads mapped on it using BWA [97] (v0.7.15; parameters: mem, -t 32). SAM output from BWA was converted into BAM, sorted, indexed, and counted using the view, sort, index, and idxstats programs, respectively, from the SAMtools software. The resulted sorted BAM files were used as input for MELT (parameters: -d 10000). Since the reads length differs between the two set of libraries (150 bp for gonads and 260 bp for optic lobe), the optic lobe dataset was trimmed with Trimmomatic (v0.32; parameters: CROP:200, HEADCROP:50) to obtain homogeneous reads of 150 bp in all the datasets. We filtered the integration sites (ISs) identified by MELT for entries which passed all the MELT checks and which presented at least 3 discordant pairs of reads supporting both left and right sides of the breakpoints. BLAST (v2.6.0; parameters: -evalue 99999) search of the candidate retrotransposons consensus sequences was performed against the genome and the identified ISs were additionally filtered out when the BLAST search showed similarity in a range of 260 bp around the IS breakpoint. The same analysis was performed using non-trimmed reads and two additional ISs identification programs, RetroSeq [98] and an in-house developed pipeline, and the significance of the results was maintained (data not shown).

### Evaluation of the activity of the RTE element discovered in O. bimaculoides

We performed 30X coverage WGS of the DNA extracted from two different tissues (SUB and GILL) of two different *O. bimaculoides individuals*. About 150 ng of genomic DNA was processed in order to construct a whole-genome Illumina sequencing library using the Illumina DNA Prep kit according to the standard protocol in a manual procedure. The library QC has been performed by the Agilent DNA 1000 Kit run on the 2100 Bioanalyzer (Agilent Technologies, Inc.). We obtained 8 libraries with 62 nM average concentration and 513 bp average size. A 0.9-nM final library pool has been loaded on a NovaSeq 6000 S2 Reagent Kit (300 cycles) and run on the NovaSeq 6000 System. We obtained an average %Q30 = 90.4, 84.8% clusters passing filter, and a total output 1.55 Tb. The identification of non-reference LINE insertion events in GILL and SUB of two different individuals was performed using MELT (version 2.2.2). First, reads were mapped to the reference genome using bwa with default parameters (version 0.7.15). Then, the MEI zip file needed for the subsequent MELT analysis was generated by using the MELT BuildTransposonZIP command by (i) setting the error value to 3, (ii) providing the FASTA sequence of the RTE LINE, and (iii) providing the genomic coordinates of the annotated insertion sites of the RTE LINE (previously identified by masking the LINE sequence on the *O. bimaculoides* reference genome by RepeatMasker—version 4.0.5). Finally, MELT SPLIT analysis was run following the Preprocess, IndivAnalysis, GroupAnalysis, Genotype, and MakeVCF steps as indicated in the MELT documentation (https://melt.igs.umaryland.edu/manual.php). We selected only the integration sites which could be identified by at least 3 supporting reads and passing all the quality checks performed by the MELT (classified as PASS). The results were evaluated using the UpSetR library [99].

### Phylogenetic tree generation

Evolutionary tree in Fig. 3d was generated using 100 full-length LINEs belonging to 15 LINE clades (Additional file 1: Table S4). Protein sequences were selected from Ohshima and Okada [39] and manually checked. InterPro was used to identify endonuclease and reverse transcriptase domains in all the LINEs. Multiple sequence alignments were performed using MAFFT [100] (v7.221; with option L-INS-i). We utilized TrimAl [101] (v1.4.rev15) to perform automated trimming aligned sequences (parameters: -fasta -automated1). Phylogenetic relationships between LINE elements were reconstructed with MrBayes [102] (v3.2.1). Bayesian analysis was run for six million generations with twenty-two chains, sampling every 1000 generations (6000 samples). Convergence was attained with a standard deviation of split frequencies below 0.01 and a consensus tree was generated using a burnin parameter of 1500 (25% of 6000 samples). The phylogenetic tree was visualized with FigTree program (release 1.4.2; http://tree.bio.ed.ac.uk/software/figtree/).

### Classification of transcripts expressed in each sample, expression peaks, and selection of candidates for validations

We classified subsets of transcripts according to their expression levels across the different parts. A transcript was considered expressed in a specific part if, in all the replicates of that part, it showed an expression level > 0.5 CPM. This resulted in the classification of the groups of transcripts used to perform the analysis. We also classified transcripts having a peak of expression. These represent transcripts showing an expression level > 0.5 CPM in all three biological replicates of exclusively one part and below 0.5 in the others, which resulted in ~ 1800 transcripts. They were used to draw the heatmap using MeV (release 4.8.1) as part of the TM4 suite [103] with hierarchical clustering exploiting Pearson correlation. Candidates for validations were selected among the 1800 transcripts with expression peak using the following additional criteria. For coding transcripts, we randomly selected four coding transcripts representing octopus homologs of homeobox genes. The annotation was manually verified. For lncRNAs, we randomly selected four putative non-coding containing a SINE fragment. Both coding and lncRNA candidates were validated using RT-PCR and Sanger sequencing.

### Polymerase chain reaction (PCR)

*Octopus vulgaris* cDNAs were generated from 200 ng of total RNA using Superscript VILO cDNA Synthesis Kit (Life Technologies) in 20 μl reaction volume. PCR were carried out using 20 ng of cDNA, 0.25 μl of Taq DNA Polymerase (5 U/μl; Roche), 1 μl of each specific forward and reverse primer (25 pmol/μl), 2.5 μl of PCR reaction buffer (10×), 2.5 μl of dNTP mix (10×), and water (up to final volume 25 μl). The ubiquitin gene (accession number FJ617440) was used as an internal control. Reactions for the coding transcripts were amplified with a single step of 2 min at 94 °C, 15 s at 94 °C, 30s at 60 °C, and 1 min at 72 °C for 35 cycles and 7 min for 72 °C. Reactions for the non-coding transcripts were amplified at an annealing temperature of 58 °C. The following primers were utilized:

- Arx forward 5′-TCCCTGCCTTCTCAACACAT-3′
- Arx reverse 5′-TCCGAACTTCCACGCTTACT-3′
- Hoxb5a forward 5′-GTGGCGAGGAATTTAGGAAG-3′
- Hoxb5a reverse 5′-GCAACAGTCATAGTCCGAACAG-3′
- Phox2b forward 5′-AATGGGGTGAGATCCTTTCC-3′
- Phox2b reverse 5′-TTCATTGCAATCTCCTCTCG-3′
- Meox2 forward 5′-TCCAGAACCGTCGGATGAAA-3′
- Meox2 reverse 5′-TACGTAAAGGGCACACACCT-3′
- Seml forward 5′-CACTTGTGCAAGGTACCACG-3′
- Seml reverse 5′-AGGTCTCCTTAAATTTATTTCTGTGCA-3′
- Subl forward 5′-ACAGAGCATCTTGAGTCTCACT-3′
- Subl reverse 5′-CACTCCTGCGCCTTTCATTT-3′
- Oll forward 5′-GGATTGACCCTGCAACTTGG-3′
- Oll reverse 5′-CAGTGATGACGGACTTGCAA-3′
- Arml forward 5′-GTACCCCACAAAATTAAATC-3′
- Arml reverse 5′-CACTCACAAGGCTTTAGTTGGC-3′
- Ubi forward 5′-TGTCAAGGCAAAGATTCAAGA-3′
- Ubi reverse 5′-GGCCATAAACACACCAGCTC-3′

### Cloning and primer walking of the LINE element in Octopus vulgaris

PCR has been carried out on cDNA and gDNA with Takara LA Taq and the primer pair Line F - Line R7 with the following amplification program: 1 min at 94 °C (30 s at 94 °C, 5 min at 68 °C) × 35 cycles, 10 s at 72 °C. The specific amplicon obtained on cDNA has been gel extracted and cloned in pGEM – T Easy Vector System following manufacturer instructions. The cDNA clone has been Sanger sequenced with the following primers:

- SP6 (pGEM’s multiple cloning region) 5′-ATTTAGGTGACACTATAGAA-3′
- LineF 5′-CCCCAGTCGTCTTGACTTTG-3′
- LineF1 5′-GAGCAGCCCTCTTCAGGAT-3′
- LineF2 5′-GCGACCATCATCAGTGCTTA-3′
- LineR2 5′-TCAGATGCCAGTGTTTGGAG-3′
- LineF3 5′-GGGTCAGAAAGTGACGAGGA-3′
- LineR3 5′-TGCATGAGGCGGAGTTTAG-3′
- LineF4 5′-CAAGAGGCTGATCCTGGAGA-3′
- LineR4 5′-CCGATCTCCTTTCCGCTTAT-3′
- LineF5 5′-AGGAGAAATGCATGGAGCAG-3′
- LineR5 5′-TGTTGATACCGGACTTGCAG-3′
- LineR6 5′-CGGTAAGCAGTCCACGTCTC-3′
- LineR7 5′-GAACTGCCGCCATGAGAC-3′
- T7 (pGEM’s multiple cloning region) 5′-TAATACGACTCACTATAGGG-3′

### LINE copy number variation using quantitative real-time PCR in *Octopus vulgaris*

Copy number variation analysis was performed on genomic DNA extracted from octopuses (*N* = 9; SEM, SUB, OL, and ARM). One ARM sample was chosen as calibrator, while 18S was chosen as invariant control. Purified genomic DNA concentrations were assessed by NanoDROP (Thermo Fisher Scientific). According to the starting concentration, DNA samples were diluted in TE buffer (10mM Tris-HCl, 1mM EDTA, pH 7.5) to a concentration of 100 ng/μL and then further diluted to a concentration of 10 ng/μL. All dilutions were checked by NanoDROP. Primers and multiplexing efficiencies were verified by linear regression to a standard curve ranging from 50 ng to 16 pg of genomic DNA. LINE and 18S slopes were − 3.3 and 3.8, respectively, and represent acceptable amplification efficiencies. Standard curves also confirmed that the final concentration of 5 ng DNA tested in qPCR was within the linear range of reaction. Reactions were performed in 20 μl reaction mixture containing iQ Multiplex Powermix (Bio-Rad), Taqman primers (10 μM), and probes (10 μM) differentially labeled (with FAM or VIC fluorophore) and specifically designed to hybridize with the target DNA sequences. LINE element was amplified using the following primers:

- LINE forward 5′-AGCAGTGGGAATCATTCA-3′
- LINE reverse 5′-GTCGTTTTCGTCGAACCAGT-3′

18S was amplified using the following primers:

- 18S forward 5′-AGTTCCGACCGTAAACGATG-3′
- 18S reverse 5′-CCCTTCCGTCAATTCCTTTA-3′

As probe sequences we utilized the following:

- FAM: 5′-AACTCTGGGCCAAATTACGA-3′
- VIC: 5′-GGGAAACCATAGTCGGTTCC-3′

qPCR was carried out for 20 s at 90 °C, followed by 40 cycles of 10 s at 95 °C and 30 s at 59 °C using the 7900HT Fast Real Time PCR System (Applied Biosystems). Assays were performed for each sample in duplicate and reproduced four times. Data obtained from the co-amplifications of the target DNA sequence and the internal invariable control 18S were analyzed using the 2–ΔΔCt method79.

### Southern blotting

The brains (SEM, SUB, and OL) and a piece of an arm (ARM) of three *O. vulgaris* were dissected after humane-killing and immediately frozen in liquid nitrogen. Pulverized samples were treated following the methods utilized by Perelman and coworkers80; in brief, after phenol∶chloroform (50:50) extraction DNA was precipitated using cold isopropanol followed by centrifugation, suspended in TE buffer (10 mM Tris–HCl pH 8.0 and 0.1 mM EDTA pH 8.0), treated with ribonuclease A (10 μg/mL) and incubated at 37 °C for 30 min. DNA concentration was estimated using NanoDrop and quality checked by electrophoresis on 0.8% agarose gel. 10 μg genomic DNA for each tissue was digested with EcoRI (New England Biolabs) overnight at 37 °C and resolved on a 0.9% agarose gel for 15 h at 1.5V. DNA was transferred to a Hybond-N+ nylon membrane (0.45 μm; Amersham Pharmacia Biotech) according to Sambrook and Russell81. DIG-labeled LINE DNA probe was prepared by PCR DIG Probe synthesis kit (Roche). Hybridization and autoradiography were performed according to the DIG Application Manual (Roche).

### Probe synthesis for in situ hybridization

We amplified by PCR a 356-bp cDNA fragment of the assembled LINE from bp 1512 to bp 1868 using the following primers:

- LINE forward 5′-GGGTCAGAAAGTGACGAGGA-3′
- LINE reverse 5′-TGCATGAGGCGGAGTTTAG-3′

The choice of the fragment and the design of primers have been based on manual curation steps ensuring that the chosen fragment is present exclusively in the transcript of the identified LINE element and in no other assembled transcripts. The amplified fragment was cloned into TOPO® TA Cloning® vector (Life Technologies, CA, USA) according to the manufacturer’s protocol. Cloned fragment was digested using BAMHI and ECORV restriction enzymes and validated by Sanger sequencing. Sense and antisense digoxigenin-labeled RNA probes were generated by in vitro transcription using the DIG-RNA Labeling Kit (SP6/T7; Roche Applied Sciences, QC, Canada). Labeled RNA probes were quantified by dot blot analysis.

### In situ hybridization experiments

Brain masses (SEM, SUB, OL) and a segment of an arm from octopuses (*N* = 3) were fixed in paraformaldehyde 4% (PFA) in phosphate-buffered saline (PBS) at 4 °C (3h for brain masses; overnight, ARM). Samples were washed (four rinses in PBS), dehydrated in series of graded methanol/PBS (1:3, 1:1, 3:1 v/v), and stored at least one night in methanol (− 20 °C). Tissues were then rehydrated at 25 °C in a series of graded methanol/PBS (3:1, 1:1, 1:3 v/v) solutions and cryoprotected in 30% sucrose in PBS. After sucrose infiltration, samples were embedded in tissue freezing medium (OCT; Leica Biosystems) and sectioned using a cryostat (Leica CM3050 S). Sagittal and/or coronal sections (40 μm) were collected in PBST (phosphate-buffered saline including 0.1% Tween™ 20 and 0.2mM sodium azide). Washed free-floating sections were mounted on Superfrost Plus slides (Menzel Gläser) and let dry overnight under fume hood. Hybridizations were performed as described by Abler et al. 82 with modifications. After rehydration in PSBT, the sections were quenched at 25 °C in 6% H_2_O_2_ (30 min) treated with proteinase K (10 min) and post-fixed with PFA-G (4% paraformaldehyde and 0.2% glutaraldehyde) for 20 min. Prehybridization step was performed in hybridization solution (HB 50% formamide, 5× SSC, with 10 μg/mL heparin, 10 μg/mL yeast tRNA, and 1% Blocking reagent) for at least 1 h at 60 °C and then incubated overnight in HB with the digoxigenin-labeled riboprobes. Post-hybridization washes (50% formamide, 5× SSC, 1% SDS) were carried out for 2 h at 60 °C. The sections were washed in TNT (10mM Tris-HCL pH 7.5, 0.5 M NaCl, 0.1% Tween™ 20) at 25 °C and incubated for 15 min at 37 °C with RNase (0.25 μg/mL), followed by a FS (50% formamide, 5× SSC) incubation of 2 h (60 °C). DIG was detected with an alkaline phosphatase labeled antibody (Roche). After a saturation step in TBS pH 7.5 (10% sheep serum, 1% blocking reagent, 1% BSA, 0.1% Tween™ 20) for 1 h (room temperature), the sections were incubated overnight at 4 °C with antibodies (1:1000; in TBS containing: 5% sheep serum, 1% blocking reagent, 1% BSA). The following day, sections were washed for 2 h in TBS (pH 7.5; 0.1% Tween™ 20 and 2 mM levamisole) and then washed in alkaline phosphatase solution (100 mM Tris-HCL pH 9.5, 100 mM NaCl, 50 mM MgCl_2_, 0.1% Tween™ 20 and 2 mM levamisole). Bound antibodies were revealed using NBT-BCIP (Roche). After DIG in situ hybridization, slides were counterstained with DAPI (5 μg/mL, Invitrogen) washed and mounted using aqueous mounting.

### RTE-2_OV custom-made polyclonal antibodies

Custom-made polyclonal antibodies were obtained from Primm Biotech Custom Antibody Services (Milan, Italy) and raised against two peptides derived from two portions of the RTE-2_OV protein: GAA (1-100 aa) and HAA (569-673 aa) resulting to be unique within the translation of the assembled transcriptome. To choose the portion to select, the manufacturer also took into consideration protein similarity (selection of regions with no significant identity to the murine and rabbit proteome), low complexity and transmembrane regions (exclusion of such regions), and distribution of predicted antigenic peptides (selection of regions with a high number of predicted antigenic peptide). The selected synthetic peptides were injected into two rabbits and boosted three times within 38 days (at days 21, 28, 35) after the first injection. The final bleeding was conducted 3 days after the last injection, and the crude sera were purified on Sepharose columns by immunoaffinity.

### Immunohistochemistry and antibody validation

SEM, SUB, and OL dissected from *O. vulgaris* (*N* = 3) were immediately immersed 4% paraformaldehyde (PFA) in seawater (4 °C for 3 h). After fixation, samples were washed several times in PBS, cryoprotected in sucrose 30%, and embedded in OCT compound (OCT; Leica Biosystems). The embedded brain parts were then sectioned at 20 μm using a cryostat (Leica CM3050 S). No antigen retrieval was required. Tissue sections were rehydrated in three successive baths of 0.1 M PBS and incubated for 90 min (at RT) in 5% goat serum (Vector Laboratories Ltd.) diluted in 0.1 M PBS containing 0.05% Tween (PBTw). The slices were subsequently incubated at 4 °C with custom-made polyclonal antibodies raised against LINE (G and H; see RTE-2_OV custom-made polyclonal antibodies for details). The next day, slices were again washed by several changes of PBTw and incubation (at RT for 90 min) with secondary antibodies was carried out using Alexa Fluor 488 or 546 goat anti-rabbit IgG both diluted 1:200 in PBTw. Subsequently, sections were rinsed, and the cell nuclei were counterstained with DAPI (Molecular Probes, Eugene, OR). Finally, after further extensive washes, the sections were mounted with fluorescent mounting medium (Fluoromount, Sigma). For all antisera tested, omission of the primary antiserum and/or secondary antiserum resulted in negative staining. In addition, specificity was assured by pre-incubating (4 °C, overnight) the antibodies with 1 mg/mL of synthetic epitope (HAA and GAA, see RTE-2_OV custom-made polyclonal antibodies for details) before staining. Again, no immunostaining was observed. The two custom-made polyclonal antibodies raised against two different peptides of the RTE-2_OV protein stained the same spatial arrangement in the octopus brain tissue.

### Imaging

Sections were observed under microscopes depending on the techniqueImage acquisition and processing were performed using the Leica Application Suite software (Leica Microsystems). For IHS, we utilized a Leica DMI6000 B inverted microscope, and for IHC, a Leica TCS SP8 X confocal microscope (Leica Microsystems, Germany). Tile Z-stacks were performed using a 0.2-μm step size. IHC figures have been assembled following guidelines for color blindness provided by Wong [104].

## Supplementary Information

**Additional file 1: Table S1.** *Octopus vulgaris* samples utilized for sequencing. **Table S2.** Counts of the assembled *Octopus vulgaris* and *O. bimaculoides* transcriptomes. **Table S3.** Counts of the repeats composition for the assembled *Octopus vulgaris* transcriptome. **Table S4.** LINEs used for phylogenetic analysis. **Fig. S1.** Completeness of Octopus transcriptomes. **Fig. S2.** Stringent classification of lncRNAs. **Fig. S3.** Most represented GO Molecular Function and Cellular Component classes. **Fig. S4.** Percentages of transcripts associated to transposable elements. **Fig. S5.** Southern blot analysis of the *Octopus vulgaris* LINE element. **Fig. S6.** *In situ* hybridization for LINE in the brain (SEM, SUB and OL) and arm of adult *Octopus vulgaris*, and relative controls.

**Additional file 2: **Fasta file containing the *Octopus vulgaris* assembled and filtered transcriptome made up by 64,477 sequences.

**Additional file 3: **Tab delimited text file containing raw reads counts for each of the assembled *Octopus vulgaris* transcript.

**Additional file 4: **Tab delimited text file containing the annotations of the 64,477 filtered *Octopus vulgaris* transcripts.

**Additional file 5: **GFF3 file containing the mapping of the *Octopus vulgaris* transcripts on the genome.

**Additional file 6: **Fasta file containing the *Octopus bimaculoides* assembled and filtered transcriptome made up by 92,820 sequences.

**Additional file 7: **GFF3 file containing the mapping of the *Octopus bimaculoides* transcripts on the genome.

**Additional file 8: **Tab delimited text file containing the annotations of the 92,820 *Octopus bimaculoides* transcripts.

**Additional file 9: **RepeatMasker analysis output file on the repeats content of the *Octopus vulgaris* assembled transcritome.

**Additional file 10: **VCF file containing the output of MELT SPLIT run on the WGS of the *Octopus bimaculoides* samples.

## Data Availability

The RNAseq dataset of *Octopus vulgaris* supporting the conclusions of this article is available in the ArrayExpress database (www.ebi.ac.uk/arrayexpress) under accession number E-MTAB-3957. The WGS DNAseq dataset of *Octopus bimaculoides* supporting the conclusions of this article is available in the European Nucleotide Archive (https://www.ebi.ac.uk/ena) under accession number PRJEB51312.

## Abbreviations

TEs: Transposable elements
LINE: Long interspersed element
SINE: Short interspersed element
LTR: Long terminal repeat
LncRNAs: Long non-coding RNAs
RTE: Retrotransposon element
SEM: Supra-esophageal mass
SUB: Sub-esophageal mass
OL: Optic lobe
ARM: Arm
GO: Gene Ontology
CPM: Counts per million
RT-PCR: Reverse transcriptase polymerase chain reaction
qPCR: Quantitative polymerase chain reaction
MELT: Mobile Element Locator Tool
IS: Integration site
ISH: In situ hybridization
IHC: Immunohistochemistry
RBP: Ribonucleoprotein
