# Supplementary material for: Identification of LINE retrotransposons and long non-coding RNAs expressed in the octopus brain
Source: BMC Biol. 2022 May 18;20:116. doi: 10.1186/s12915-022-01303-5 (PMC9115989; doi:10.1186/s12915-022-01303-5)
Supplement: Supplementary file 1 — Additional file 1: Table S1. Octopus vulgaris samples utilized for sequencing. Table S2. Counts of the assembled Octopus vulgaris and O. bimaculoides transcriptomes. Table S3. Counts of the repeats composition for the assembled Octopus vulgaris transcriptome. Table S4. LINEs used for phylogenetic analysis. Fig. S1. Completeness of Octopus transcriptomes. Fig. S2. Stringent classification of lncRNAs. Fig. S3. Most represented GO Molecular Function and Cellular Component classes. Fig. S4. Percentages of transcripts associated to transposable elements. Fig. S5. Southern blot analysis of the Octopus vulgaris LINE element. Fig. S6. In situ hybridization for LINE in the brain (SEM, SUB and OL) and arm of adult Octopus vulgaris, and relative controls. [file 12915_2022_1303_MOESM1_ESM.pdf]

SUPPLEMENTAL FIGURES AND TABLES

Identification of LINE retrotransposons and long non-coding RNAs expressed in the octopus brain

Giuseppe Petrosino[1,*], Giovanna Ponte[1,*], Massimiliano Volpe[1,*], Ilaria Zarrella[1], Federico Ansaloni[8,2], Concetta Langella[1], Giulia Di Cristina[1], Sara Finaurini[2], Monia T. Russo[3], Swaraj Basu[1], Francesco Musacchia[1], Filomena Ristoratore[1], Dinko Pavlinic[4], Vladimir Benes[4], Maria I. Ferrante[3], Caroline Albertin[5], Oleg Simakov[6,7], Stefano Gustincich[8,2], Graziano Fiorito[1,#], Remo Sanges[1,2,8,#]

[1] Department of Biology and Evolution of Marine Organisms, Stazione Zoologica Anton Dohrn, Villa Comunale, 80121 Napoli (SZN), Italy.

[2] Neurobiology Sector, Scuola Internazionale Superiore di Studi Avanzati (SISSA), Via Bonomea 265, 34136 Trieste, Italy.

[3] Department of Integrative Marine Ecology, Stazione Zoologica Anton Dohrn, Villa Comunale, 80121 Napoli (SZN), Italy.

[4] Scientific Core Facilities & Technologies, GeneCore, European Molecular Biology Laboratory (EMBL), Meyerhofstrasse 1, 69117 Heidelberg, Germany.

[5] Marine Biological Laboratory (MBL), Woods Hole, Massachusetts, USA.

[6] Okinawa Institute of Science and Technology Graduate University, Onna, Okinawa 9040495, Japan.

[7] Department of Molecular Evolution and Development, Wien University, Althanstraße 14 (UZA I), 1090 Wien, Austria.

[8] Central RNA Laboratory, Istituto Italiano di Tecnologia (IIT), Via Enrico Melen 83, 16152 Genova, Italy.

* These authors contributed equally to this work

# corresponding authors:

Remo Sanges, e-mail: remo.sanges@gmail.com

Graziano Fiorito, e-mail: graziano.fiorito@szn.it

Table S1: *Octopus vulgaris* samples utilized for sequencing. Brain: supra-oesophageal (SEM) and sub-oesophageal (SUB) masses, and optic lobe (OL); arm: only anterior second left arm (ARM).

**Animal**	**Sex**	**Weight**	**Part**	**Partname**	**Samplename**
13/01	F	296	supra-oesophageal mass	SEM	IZ10
13/01	F	296	sub-oesophageal mass	SUB	IZ11
13/01	F	296	optic lobe	OL	IZ12
13/01	F	296	anterior arm	ARM	IZ13
13/02	M	336	supra-oesophageal mass	SEM	IZ15
13/02	M	336	sub-oesophageal mass	SUB	IZ16
13/02	M	336	optic lobe	OL	IZ17
13/02	M	336	anterior arm	ARM	IZ18
13/03	M	288	supra-oesophageal mass	SEM	IZ20
13/03	M	288	sub-oesophageal mass	SUB	IZ21
13/03	M	288	optic lobe	OL	IZ22
13/03	M	288	anterior arm	ARM	IZ23

Table S2: Counts of the assembled *Octopus vulgaris* and *O. bimaculoides* transcriptomes.

	O. vulgaris	***O. bimaculoides***
Assembled and filtered transcripts	64477	92820
Total bases	84399088	102485827
GC content (%)	37.9	37
Contig N50	2087	1573
Median transcript length (bp)	795	744
Average transcript length (bp)	1308	1104
Minimum length (bp)	201	201
Maximum length (bp)	20031	32440
248 CEGs Complete (%)	97.20	98.79
248 CEGs Complete + Partial (%)	98.4	100

Table S3: Counts of the repeats composition for the assembled *Octopus vulgaris* transcriptome.

	Nucleotides	Percentage	Transcripts	Percentage
**Bases Masked**	6584938	7.8	46944	72.8
**Retroelements**	2102784	2.5	16926	26.2
**DNA transposons**	1501995	1.8	11913	18.5
**Unclassified**	169576	0.2	250	0.4
**Total interspersed repeats**	3774355	4.5	22915	35.5
**Satellites**	150431	0.2	1326	2.1
**Simple repeats**	2368596	2.8	34833	54
**Low complexity**	470441	0.6	7704	11.9

Table S4: LINEs used for phylogenetic analysis (see also: Ohshima and Okada[36] and Jurka et al.[71]).

**Clade**	**Name**	**Species**	**Sequence ID**
CRE	SLACS	*Trypanosoma brucei*	CAA34931
	CZAR	*Trypanosoma cruzi*	AAA30239
	CRE1	*Crithidia fasciculata*	AAA75435
	CRE2	*Crithidia fasciculata*	AAB40036
Genie/Gil	GilM	*Giardia intestinalis*	AAL47180
R4	R4Al	*Ascaris lumbricoides*	AAA97394
R1	R1Dm	*Drosophila melanogaster*	CAA36227
	R1	*Bradysia coprophila*	AAA29813
	RT1	*Anopheles gambiae*	AAA29363
	RT2	*Anopheles gambiae*	AAA29365
	R1Bm	*Bombyx mori*	AAC13649
	TRAS1	*Bombyx mori*	BAA07467
	SART1	*Bombyx mori*	BAA19776
LOA	LOA	*Drosophila silvestris*	AAB22452
	BAGGINS1	*Drosophila melanogaster*	Repbase
	Bilbo	*Drosophila subobscura*	AAB92389
	Lian-Aa1	*Aedes aegypti*	AAB65093
Tad1	Tad1	*Neurospora crassa*	AAA21781
	MgL	*Magnaporthe grisea*	AAB71689
	CgT1	*Glomerella cingulata*	AAA85636
Jockey	BMC1	*Bombyx mori*	BAB21761
	amy	*Bombyx mori*	AAA17752
	Juan-A	*Aedes aegypti*	AAA29354
	Juan-C	*Culex pipiens*	AAA28291
	NLR1Cth	*Chironomus thummi*	AAB26437
	Doc6	*Drosophila melanogaster*	Repbase
	G5	*Drosophila melanogaster*	Repbase
	G5A	*Drosophila melanogaster*	Repbase
	Jockey	*Drosophila melanogaster*	AAA28675
	Doc	*Drosophila melanogaster*	CAA35587
	Fw	*Drosophila melanogaster*	AAA28508
	Fw2	*Drosophila melanogaster*	Repbase
	G4	*Drosophila melanogaster*	Repbase
	Helena	*Drosophila yakuba*	AAC24972
	BS	*Drosophila melanogaster*	Repbase
	BS3	*Drosophila melanogaster*	Repbase
	TART-B1	*Drosophila melanogaster*	AAC46494
I	I-1_DR	*Danio rerio*	Repbase
	IVK	*Drosophila melanogaster*	Repbase
	I	*Drosophila melanogaster*	AAA70222
	ingi	*Trypanosoma brucei*	CAA29181
	L1Tc	*Trypanosoma cruzi*	CAB41693
Rex1	Rex1-1_DR	*Danio rerio*	Repbase
CR1	Sam3	*Caenorhabditis elegans*	AAA93347
	Sam1	*Caenorhabditis elegans*	AAA21080
	Q	*Anopheles gambiae*	AAA53489
	CR1-3_AG	*Anopheles gambiae*	Repbase
	CR1-5_AG	*Anopheles gambiae*	Repbase
	T1	*Anopheles gambiae*	AAA29367
	CR1-2_AG	*Anopheles gambiae*	Repbase
	CR1-4_AG	*Anopheles gambiae*	Repbase
	DMCR1A	*Drosophila melanogaster*	Repbase
	CR1	*Gallus gallus*	AAC60281
	PsCR1	*Platemys spixii*	BAA88337
	L3	*Homo sapiens*	Repbase
	SR1	*Schistosoma mansoni*	AAC06263
L2	UnaL2	*Anguilla japonica*	Repbase
	Maui	*Takifugu rubripes*	AAD19348
	CR1-3_DR	*Danio rerio*	Repbase
	CR1-1_AG	*Anopheles gambiae*	Repbase
R2	R2Bm	*Bombyx mori*	AAB59214

Clade	Name	Species	Sequence ID
	R2Nv	*Nasonia vitripennis*	AAC34927
	R2Fa	*Forficula auricularia*	AAC34906
	R2Dm	*Drosophila melanogaster*	CAA36225
	R2Am	*Anurida maritima*	AAC34903
	R2Lp	*Limulus polyphemus*	AAC34904
NeSL-1	NeSL-1	*Caenorhabditis elegans*	CAB04870
RTE	BovB	*Bos taurus*	Repbase
	BovB_VA	*Vipera ammodytes*	Repbase
	RTE-1	*Caenorhabditis elegans*	AAA50641
	RTE-2	*Caenorhabditis elegans*	AAB00700
	RTE-1_AG	*Anopheles gambiae*	Repbase
	JAM1	*Aedes aegypti*	Repbase
	Rex3	*Xiphophorus maculatus*	Repbase
	SR2	*Schistosoma mansoni*	Repbase
	RTE-3_AG	*Anopheles gambiae*	Repbase
	RTE-2_CPB	*Chrysemys picta bellii*	Repbase
	RTE-4_AMi	*Crocodylidae*	Repbase
	RTE-5_AMi	*Crocodylidae*	Repbase
	RTE-6_AMi	*Crocodylidae*	Repbase
	RTE-7_AMi	*Crocodylidae*	Repbase
	RTE-8_AMi	*Crocodylidae*	Repbase
L1	L1Hs	*Homo sapiens*	AAA51622
	L1Md	*Mus musculus*	AAA66024
	L1-1_DR	*Danio rerio*	Repbase
	L1-10_DR	*Danio rerio*	Repbase
	L1-6_DR	*Danio rerio*	Repbase
	L1-8_DR	*Danio rerio*	Repbase
	L1-3_DR	*Danio rerio*	Repbase
	L1-5_DR	*Danio rerio*	Repbase
	L1-2_DR	*Danio rerio*	Repbase
	L1-4_DR	*Danio rerio*	Repbase
	DRE	*Dictyostelium discoideum*	Repbase
	ATLINE1_4	*Arabidopsis thaliana*	Repbase
	ATLINE1_5	*Arabidopsis thaliana*	Repbase
	Tal1-1	*Arabidopsis thaliana*	AAA75254
	ATLINE1_6	*Arabidopsis thaliana*	Repbase
	Cin4	*Zea mays*	Repbase
	Tx1	*Xenopus laevis*	AAA49976
	Zepp	*Chlorella vulgari*	BAA25763

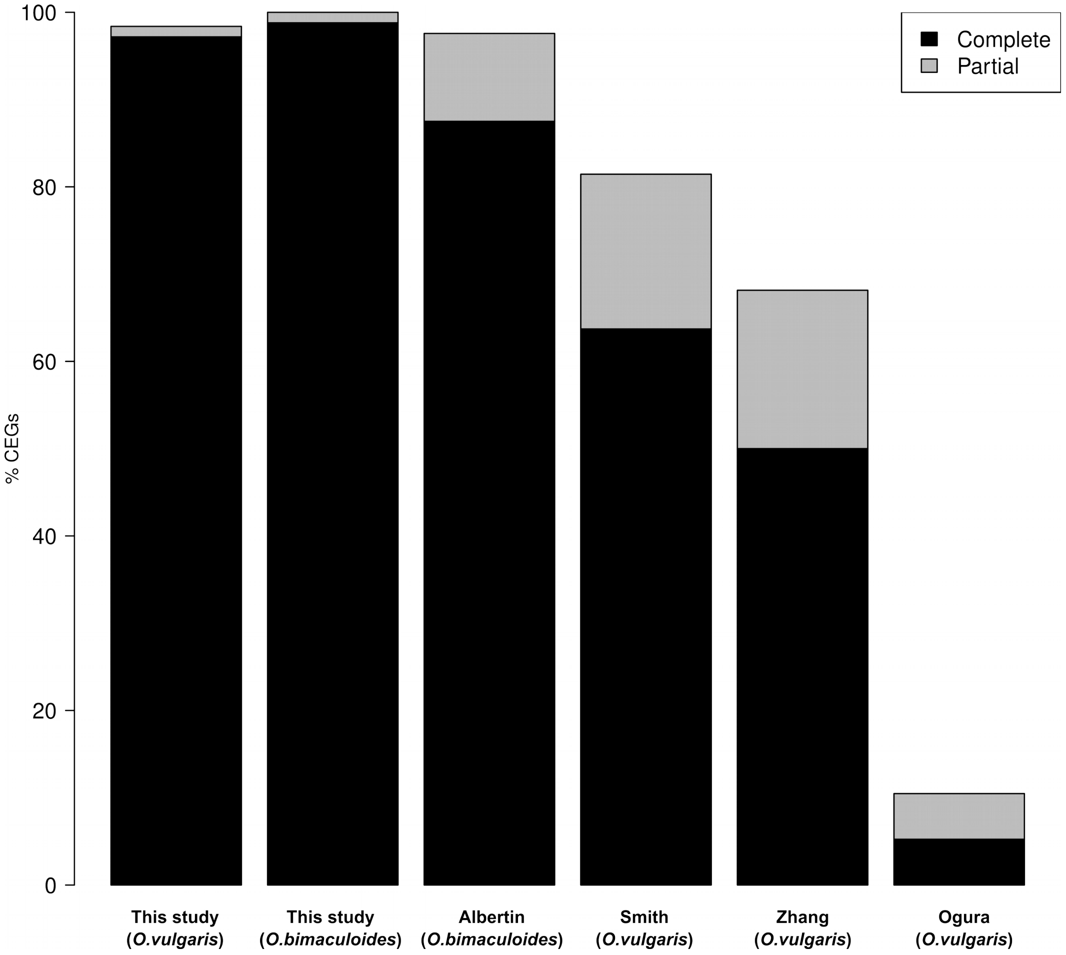

Figure S1: Completeness of *Octopus* transcriptomes. Percentages of core eukaryotic genes (CEGs) represented into this and published assemblies of octopus transcriptomes (Albertin et al.[5]; Smith et al.[62]; Zhang et al.[84]; Ogura et al.[85]). The barplots indicate the percentages of CEGs present into every published transcriptome. The portion defined as "complete" identify all those transcripts whose assembled sequence are predicted to be full-length while "partial" indicates the reconstruction of only a fragment of specific CEGs. All the transcriptomes originate from *Octopus vulgaris* with the exception of the transcriptome from Albertin et al.[5] which originate from *Octopus bimaculoides*. Two transcriptomes for *O. bimaculoides* are included here, the original one assembled by Albertin et al.[5] and the same assembled for the aims of this study as described in Methods. The transcriptomes assembled in this work should be considered the most complete available to date for the genus *Octopus*, since they contain the highest percentages of core eukaryotic genes according to CEGMA (98% for *O. vulgaris* and 100% for *O. bimaculoides*).

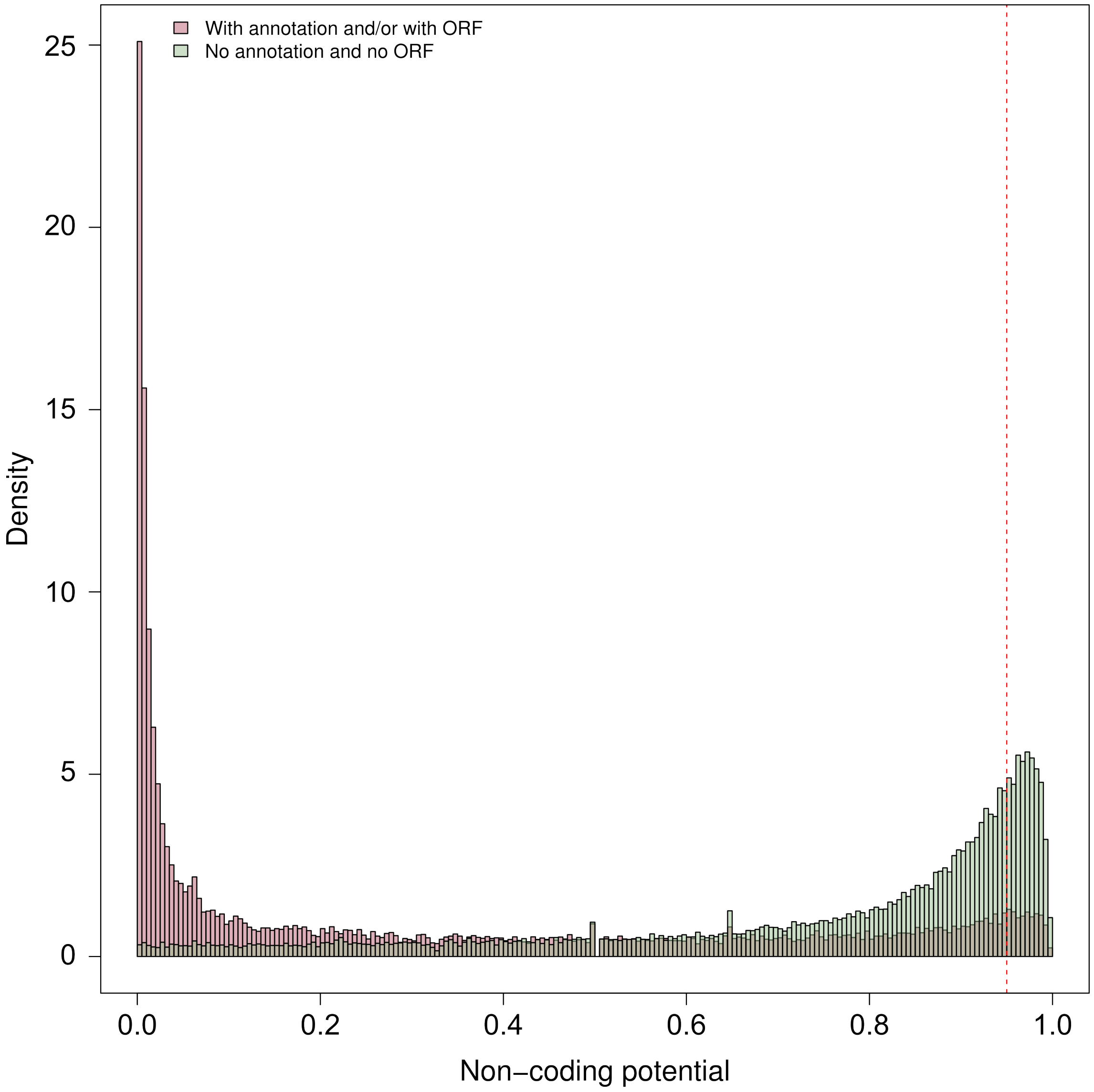

Figure S2: Stringent classification of lncRNAs. Non-coding potential score plot as measured by Portrait[57] for the assembled transcripts. The transcriptome has been divided in two groups: the first group of genes showing at least one BLAST match against a protein or a domain or a ribosomal or a small RNA in the annotation analysis and/or an ORF bigger than 100 aa (light red); and the second group of genes not showing any BLAST match and whose longest ORF results shorter than 100 aa (light green). Only transcripts without any match, with an ORF smaller than 100 aa and a non-coding potential bigger than 0.95 have been classified as non-coding. The vertical red dotted line represents the 0.95 non-coding potential cut-off used. The transcripts classified as non-coding are those plotted in the green bars at the right of the vertical red dotted line. According to Portrait recommendations, a non-coding potential score bigger than 0.5 is sufficient to classify a transcript as non-coding. We applied more stringent conditions and despite the combination of multiple parameters and the application of these stringent cutoffs we were still able to discover that a high proportion of transcripts likely represent lncRNAs. Here an underestimation of the true proportion of lncRNAs is possible because the RNAseq was not conducted using a strand specific protocol and therefore there is the possibility that annotation for "coding" has been utilized also for cases of "antisense non-coding" overlapping sense coding regions.

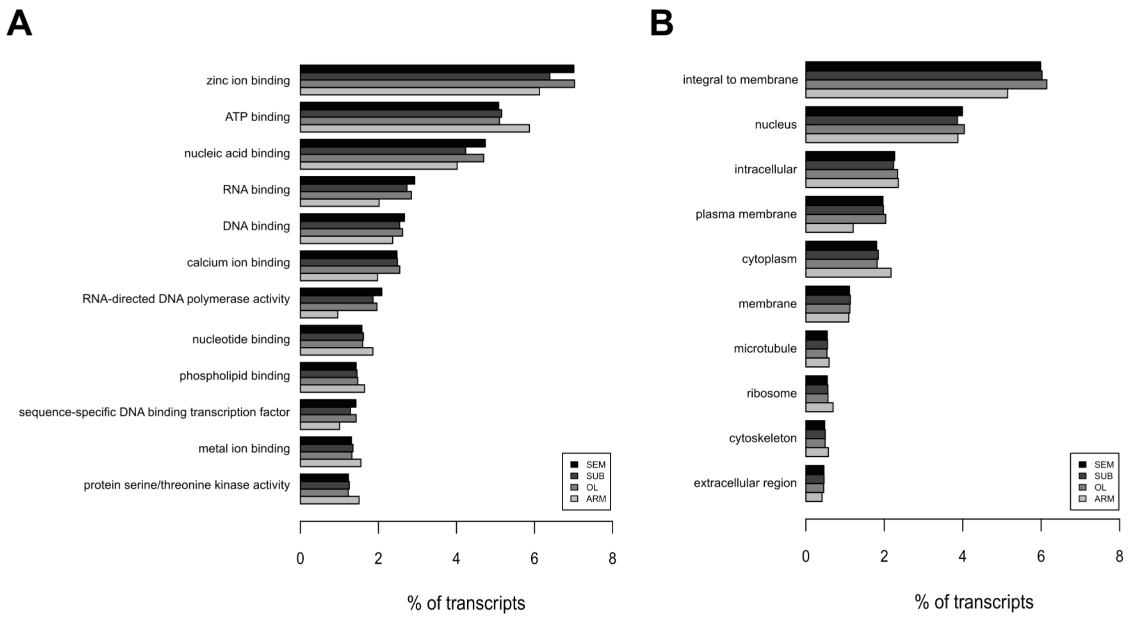

Figure S3: Most represented GO Molecular Function and Cellular Component classes. Barplots showing the percentage of top represented GO classes in the transcripts expressed for every tissue considered. The top 10 represented classes for every tissue were selected and the percentage of transcripts expressed associated to the given class is reported. **a**, Molecular function classification confirms the findings obtained with the biological process classification showing a higher rate of RNA-directed DNA polymerase activity in samples deriving from the brain. It is also important to underline that the top represented class in all the parts is the zinc ion binding which confirm the expansion of zinc fingers protein in octopus[5]. **b**, Transcriptome classification according to cellular component division results to be generally similar among the sampled parts. The octopus brain (SEM, SUB, OL) appears to contain a higher number of transcripts whose protein product is localized to the plasma membrane; higher representation of transcripts localized into the cytoplasm are found in ARM.

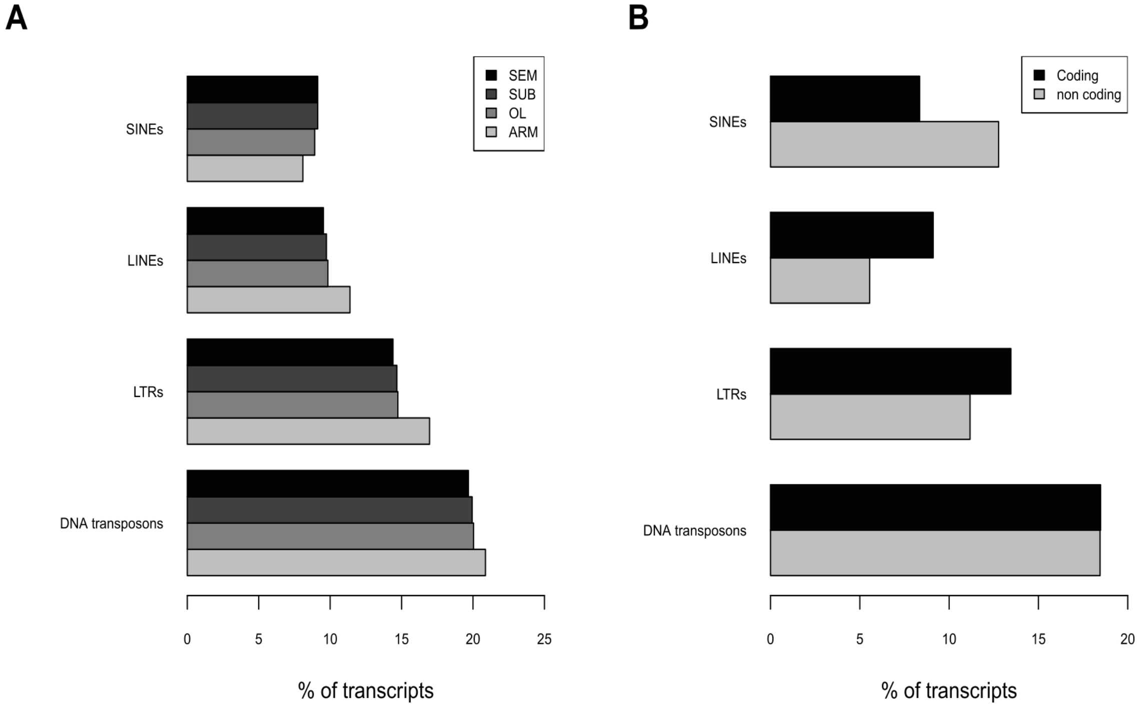

Figure S4: Percentages of transcripts associated to transposable elements. a, Barplots representing the percentage of expressed transcripts containing a fragment from a transposable element. SINE elements are most frequently embedded in brain-expressed transcripts while LINEs, LTRs and DNA transposons are associated to a higher number of arm-expressed transcripts (brain: SEM, SUB, OL; arm: ARM). **b**, Barplots representing the percentage of expressed coding and non-coding transcripts containing fragments from the different transposons. SINEs are enriched in non-coding transcripts while LINEs and LTRs fragments are more frequently embedded in coding transcripts. DNA transposons results to be equally distributed.

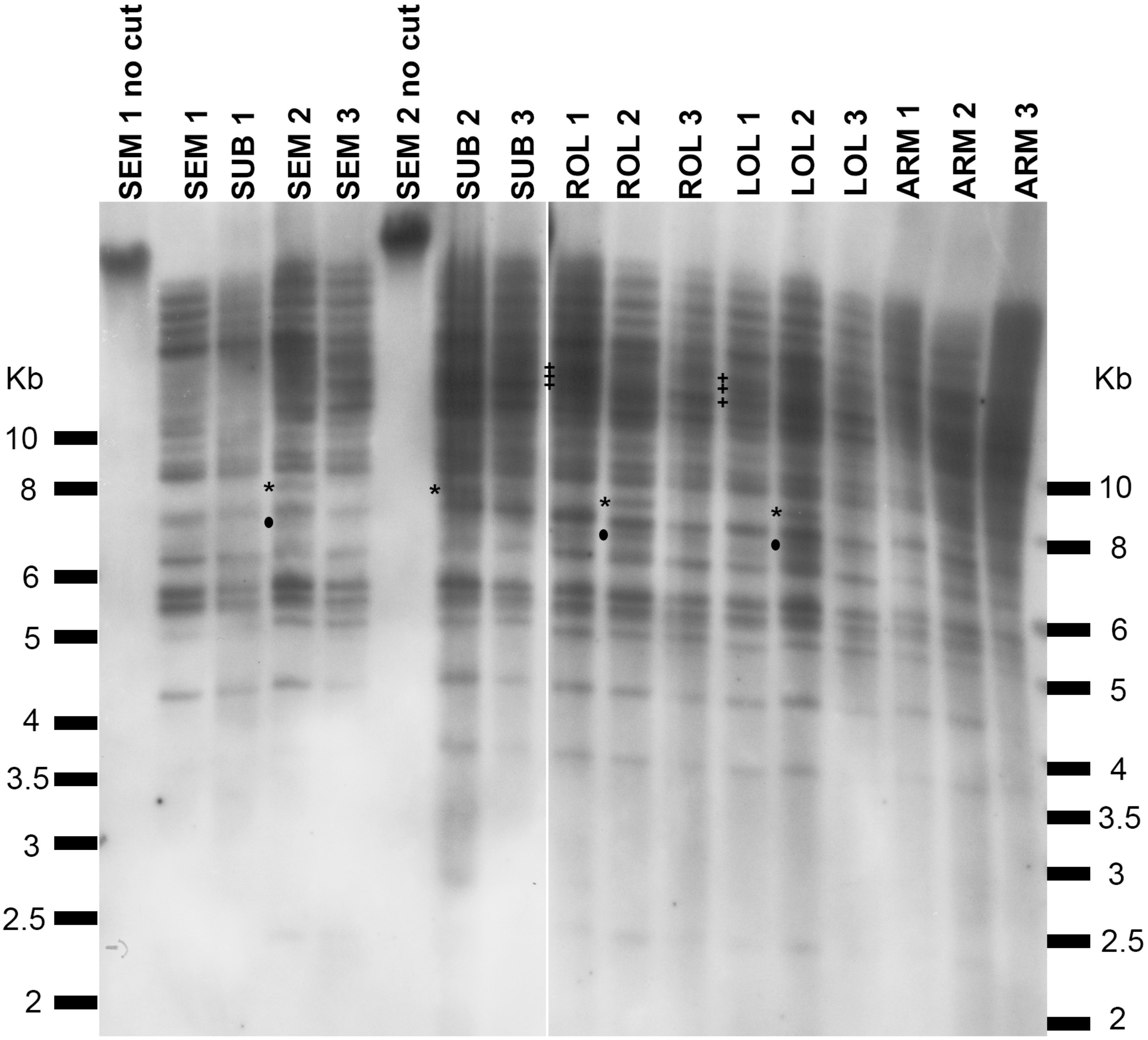

Figure S5: Southern blot analysis of the *Octopus vulgaris* LINE element. Genomic DNA extracted from SEM, SUB, OL and ARM of three different animals (#: 1, 2 and 3) were digested by restriction enzyme EcoRI and analyzed by Southern blotting. The fragment indicated by an asterisk (*) is specific of the individual #2. A plus symbol (+) highlights fragments present in OL and probably ARM of the individual #1, but not in SEM and SUB of the same individual. A dot (•) marks a fragment found in all the tissues of octopus #2 except in the SUB. DNA molecular markers are reported on both sides of the panel.

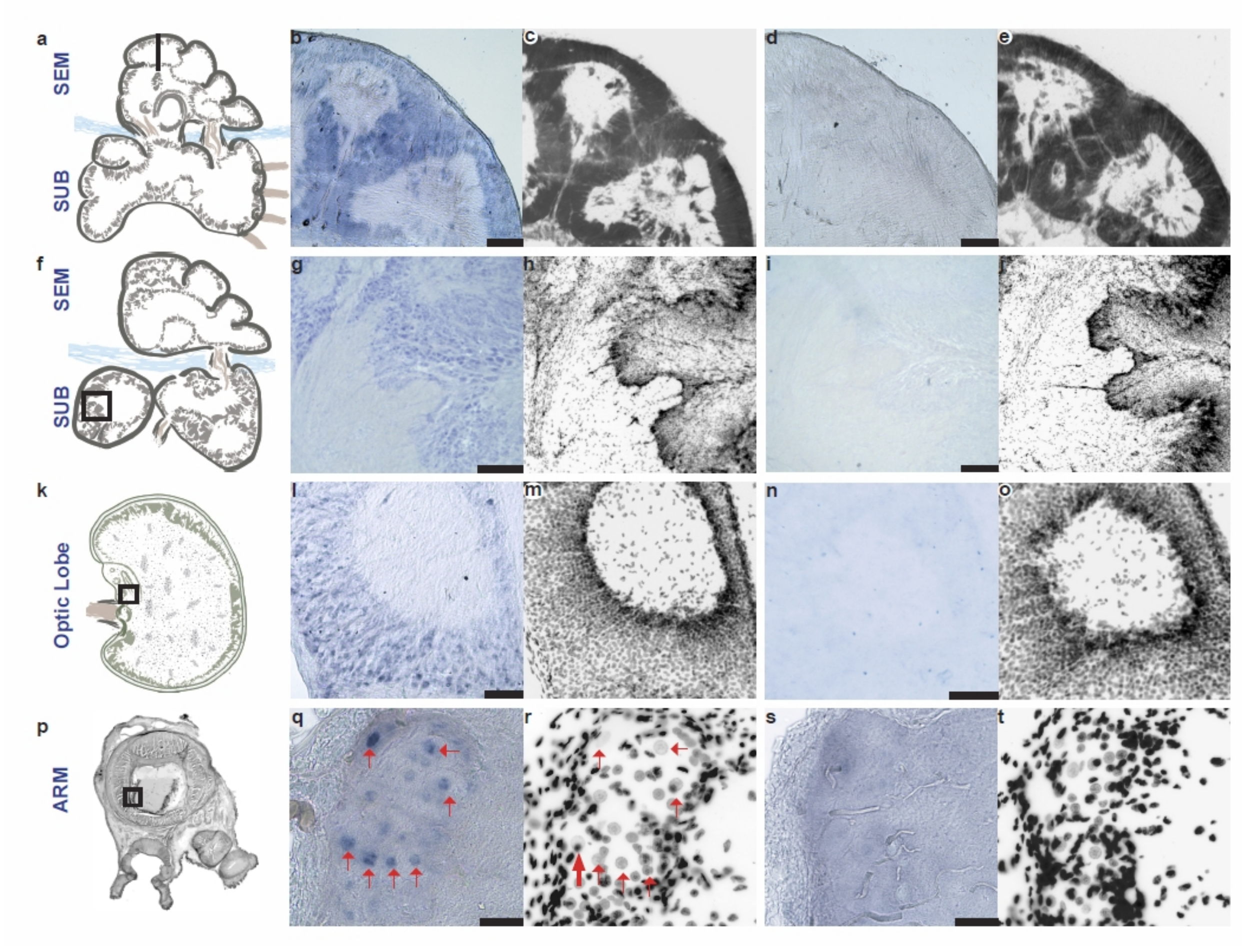

Figure S6: *In situ* hybridization for LINE in the brain (SEM, SUB and OL) and arm of adult *Octopus vulgaris,* and relative controls. Schematic diagrams (**a**, **f**, **k**, **p**) of the organization of the octopus brain (SEM and SUB) at the sagittal (**a**) and parasagittal (**f**) planes, of the optic lobe (OL, horizontal section across the midline: **k**), and of the arm (**p**) with the typical distribution of different muscular bundles surrounding the arm nerve cord, in the middle; suckers appear on the ventral side (bottom). The diagram of the octopus arm has been drawn by superimposing tracings (after Milligan staining) of a typical transverse section at the medial length of an arm. **b**, RTE-2_OV mRNA is detected in numerous cells of the vertical lobe mostly at the cortical layer of each girus. **c**, the corresponding section after DAPI staining where only nuclei appear marked. **d**,**e**, A similar section at the level of the vertical lobe after staining with sense RTE-2_OV probe serve as control (the same section is shown also after DAPI staining, **e**). **g-l**, Sections of the SUB at the level of the posterior pedal lobe, with positive cells marked after *in situ* hybridization (**g**) and the corresponding DAPI stained cells (**h**) to reveal the intricate patterns of neurons. Control staining (sense) where no positive cells are revealed are shown in (**i**) again with the same section stained to show nuclei (DAPI, **j**). **l**, An area of the peduncle lobe (OL) at the level of the spine where RTE-2_OV mRNA appear localized (the same section after DAPI, **m**). **n**,**o**, A nearby proximate section at the same level (OL) after *in situ* hybridization with sense RTE-2_OV probe (**n**) and DAPI staining (**o**). **q**, RTE-2_OV mRNA is seen at the octopus arm only in few large motor neurons (arrows) of the nerve cord; note the corresponding section after hybridization with sense probe (**s**). DAPI staining of the same sections is shown in (**r, t**). Sections stained with DAPI are presented to show the cellular populations of the respective areas. Scale bars: 100 μm.
